# Sleep disordered breathing has minimal association with retinal microvascular diameters in a non-diabetic sleep clinic cohort

**DOI:** 10.1371/journal.pone.0279306

**Published:** 2023-01-10

**Authors:** Kristina Kairaitis, Terence C. Amis, Rita Perri, Sharon Lee, Anne Drury, Christopher Lambeth, Paul Mitchell, Richard I. Lindley, John R. Wheatley

**Affiliations:** 1 Ludwig Engel Centre for Respiratory Research, The Westmead Institute for Medical Research, Westmead, New South Wales, Australia; 2 Faculty of Medicine and Health, University of Sydney, Westmead Clinical School, Westmead, New South Wales, Australia; 3 Department of Respiratory and Sleep Medicine, Westmead Hospital, Sydney, New South Wales, Australia; 4 Department of Ophthalmology, Westmead Hospital, Sydney, New South Wales, Australia; 5 Centre for Vision Research, The Westmead Institute for Medical Research, The University of Sydney, Sydney, New South Wales, Australia; 6 The George Institute for Global Health, Newtown, New South Wales, Australia; 7 Westmead Applied Research Centre, University of Sydney, Westmead, New South Wales, Australia; Charité - Universitätsmedizin Berlin, GERMANY

## Abstract

**Introduction:**

Obstructive sleep apnea (OSA) may increase stroke risk; retinal arteriolar (central retinal arteriolar equivalent, CRAE) diameter narrowing and/or retinal venular (central retinal venule equivalent, CRVE) widening may predict stroke. We examined relationships between sleep disordered breathing (SDB) and CRAE and CRVE and in a diabetes-free sleep clinic cohort.

**Methods:**

Patients for SDB assessment were recruited (Main Group, n = 264, age: 58.5 ± 8.9 yrs [mean ± SD]; males: 141) for in-laboratory polysomnography (standard metrics, eg apnea hypopnea index, AHI) and retinal photographs (evening and morning). A more severe SDB sub-group (n = 85) entered a 12-month cardiovascular risk factor minimisation (hypertension/hypercholesterolemia control; RFM) and continuous positive airway pressure (CPAP) intervention (RFM/CPAP Sub-Group); successfully completed by n = 66 (AHI = 32.4 [22.1–45.3] events/hour, median[IQR]). Univariate (Spearman’s correlation, t-test) and multiple linear regression models examined non-SDB and SDB associations with CRAE and CRVE measures.

**Results:**

*Main Group*: Evening CRAE predictors were: systolic blood pressure (0.18μm decrease per mmHg, p = 0.001), age (2.47μm decrease per decade, p = 0.012), Caucasian ethnicity (4.45 μm versus non-Caucasian, p = 0.011), height (0.24 μm decrease per cm increase, p = 0.005) and smoking history (3.08 μm increase, p = 0.052). Evening CRVE predictors were: Caucasian ethnicity (11.52 μm decrease versus non-Caucasian, p>0.001), diastolic blood pressure (0.34 μm increase in CRVE per mmHg, p = 0.001), hypertension history (6.5 μm decrease, p = 0.005), and smoking history (4.6 μm increase, p = 0.034). No SDB metric (all p>0.08) predicted CRAE or CRVE measures. *RFM/CPAP Sub-Group*: A one-unit increase in ln(AHI+1) was associated with a 0.046μm increase in CRAE (n = 85; p = 0.029). Mean evening CRAE and CRVE values did not change across the intervention (n = 66), but evening CRVE decreased ~6.0 μm for individuals with AHI >30 events/hr.

**Conclusion:**

No major SDB associations with CRAE or CRVE were identified, although the RFM/CPAP intervention reduced evening CRVE for severe OSA patients. Implications for cerebro-vascular disease risk remain uncertain.

**Trial registration:**

The protocol was registered with the Australian New Zealand Clinical Trials Registry (Trial Id: ACTRN12620000694910).

## Introduction

A number of studies have suggested that obstructive sleep apnea (OSA) increases risk for cardio- and cerebro-vascular diseases, including hypertension, coronary heart disease and stroke [1–3]. Within this context, potential linkages between OSA and large-blood vessel disease (eg carotid artery) have received particular attention [4–6], however relationships with microvascular pathology have received less attention.

There is one microvascular bed that can be readily and non-invasively accessed in clinical settings by using digital photography to provide qualitative and quantitative assessment of the retinal microcirculation [7,8]. Importantly, retinal microvasculature analysis has also emerged as a powerful and non-invasive methodology for indirect assessment of the cerebral microcirculation [8].

Large population-based studies have reported that relative narrowing of retinal arteriolar calibre, and/or widening of retinal venular calibre, carries predictive significance for hypertension, stroke and coronary heart disease [9,10], while two smaller studies have suggested an association between OSA severity and both retinal venular widening and arteriolar narrowing [11,12]. As retinal microvascular morphology may be indicative of the cumulative impacts arising from a life-long burden of vascular risk factors [13], an increase in our understanding of the contribution of OSA to these processes would have important clinical benefits for OSA patients, particularly through an enhanced ability to provide targeted, intensive, risk factor management for those at greatest vascular risk.

The aim of the present study was to conduct a cross-sectional descriptive study to determine relationships between the morphology of the retinal microvascular bed and OSA severity in a sleep clinic cohort from which the potentially confounding influence of diabetes, a known cardio-vascular risk factor, was excluded. The primary analysis focused on cross-sectional associations between CRAE and CRVE with OSA severity metrics, and included examination of relationships with overnight change (if any) in retinal vessel diameters. In addition, we undertook a 12 month Risk Factor Minimisation/CPAP (RFM/CPAP) sub-group study aimed at evaluating whether stabilising known cardiovascular disease risk factors, while concurrently treating OSA with continuous positive airway pressure (CPAP), modified retinal vessel size.

## Methods

### Registration

This study was not initially registered as a clinical trial before enrolment of participants because it was originally conceived as an observational study. The authors confirm that all onging and related trials for this intervention are registered. The protocol was registered with the Australian New Zealand Clinical Trials Registry (ANZCTR; ACTRN12620000694910).

### Study participants

We recruited 335 adults all over the age of 35 years, referred to a sleep clinic at Westmead Hospital between 2^nd^ October 2012 and 25^th^ July 2016 for investigation of potential sleep disordered breathing (SDB) and all evaluated by the same senior sleep physician (Author: KK). Informed consent was obtained, and the protocol was approved by the Sydney West Area Health Service Human Research Ethics Committee.

Participants were screened for diabetes and 40 were subsequently excluded on the basis of either a history of diabetes or a fasting blood sugar level ≥ 6.9 mmol/L, while 31 other participants either withdrew or were excluded for other reasons (e.g. failure to attend study visits, retinal photography and/or polysomnography (PSG) not performed). The remaining 264 participants formed the study “Main Group”.

Participants who were found on in-laboratory, overnight, PSG to have a Respiratory Disturbance Index (RDI) ≥ 29 events/hr, were then invited to participate in a 12-month duration cardio-vascular risk factor minimisation and CPAP intervention study between 16^th^ October 2012 and 6^th^ December 2016. A total of 85 participants (“RFM/CPAP Sub-Group”) entered this phase of the study. However, 19 subsequently withdrew or failed to complete the study, leaving 66 participants who successfully completed the 12 month intervention. Note that the RFM/CPAP Sub-group patients were recruited on the basis of RDI ≥ 29 events/hr, but for analysis we used AHI as the prime index of SDB severity to better reflect the range of hypoxic burden.

Participants in this present report also underwent carotid and femoral artery ultrasonography for measurement of intima media thickness. These results have been reported previously [14].

### Protocol

**Fig 1:**
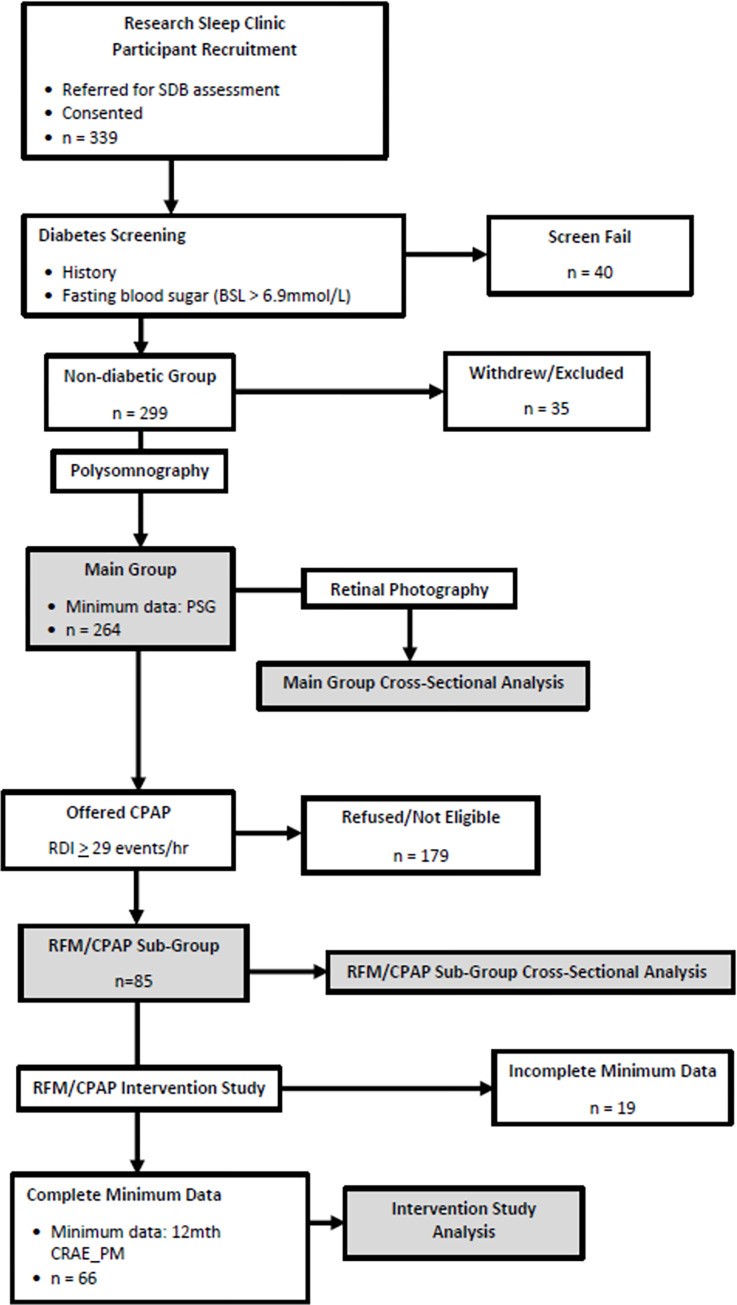
Study flow diagram. Study flow diagram showing movement of participants and data through the recruitment and screening phases, the “Main Group” and “RFM/CPAP sub-group” Cross-Sectional Analyses and the Intervention Study. SDB = sleep disordered breathing; BSL = blood sugar level; PSG = polysomnography; CPAP = continuous positive airway pressure; RDI = respiratory disturbance index; CRAE_PM = central retinal artery equivalent evening value.

Fig 1 shows a participant and data flow diagram for the study. Initial visit data included anthropometrics (height, weight, body mass index [BMI], waist circumference, hip circumference, waist hip ratio [WHR], neck circumference), a detailed medical history (including history of hypertension and hypercholesterolemia), smoking history (current or past), measurement of blood pressure (BP), fasting blood tests (total cholesterol, triglycerides, HDL cholesterol, LDL cholesterol, fasting glucose [BSL]), and an assessment of overall cardiovascular risk (Framingham Risk Score). Ethnicity was classified as Caucasian or Non-Caucasian. Sleep disordered breathing (SDB) was subsequently assessed via in-laboratory, overnight PSG. Before (evening) and after (morning) sleep retinal photographs were obtained on the PSG night.

### Polysomnography

PSG studies were recorded using Compumedics Profusion PSG4 software (Compumedics Limited; Abbotsford, Victoria, Australia) and scored according to standard guidelines [15]. The Apnea Hypopnea Index (AHI), Respiratory Disturbance Index (RDI), Oxygen Desaturation Index (ODI≥3%), Arousal Index (AI) and the percentage of sleep time spent with oxygen saturation below 90% (SaO_2_<90%) were all calculated. OSA severity was categorised by AHI as normal (AHI<5 events/hr), mild (5≤AHI<15 events/hr), moderate (15≤AHI<30 events/hr) or severe (AHI≥30 events/hr) ie a 4 category AHI analysis. For analysis purposes, OSA was additionally categorised as either non-severe (AHI<30 events/hr) or severe (AHI≥30 events/hr) ie a 2 category AHI analysis.

### Retinal photography

Retinal images were obtained from each eye with a non-mydriatic digital camera (Canon CR2-Plus, Canon, Japan) before (evening-pm) and after (morning-am) sleep, and after pupil dilation (one drop of 1% Tropicamide) in most subjects (n = 239 [pm] and n = 194 [am]). A retinal photograph centred on the optic disc was taken for each eye, with gating to diastole in most subjects (n = 242 pm and n = 238 am). Digital images were examined by a single experienced technician, using a semi-automated interactive vessel analysis (IVAN; University of Wisconsin, Madison) program. For each image, the largest six arterioles and six venules in a zone one half to one-disc diameter from the optic disc margin were measured. Average retinal arteriolar and venular width (diameter) were calculated using the Parr-Hubbard Formula and expressed as the central retinal arteriole equivalent (CRAE), central retinal venule equivalent (CRVE) and retinal arteriole:venule ratio (AVR) [16]. Data for left and right eyes were then averaged to produce a single value for each participant/condition.

### RFM/CPAP intervention (RFM/CPAP sub-group only)

After recruitment to the intervention study, participants with hypertension or hypercholesterolaemia were prescribed appropriate medication and undertook a 1-month run-in stabilisation period. Participants underwent a second PSG at Westmead Hospital Sleep Laboratory to determine the appropriate CPAP treatment pressure [17] and were then provided with a fixed pressure CPAP machine (ResMed S9; ResMed Ltd., Bella Vista, NSW, Australia) and technician fitted with an appropriate nasal mask. Over the next 12-months participants were monitored monthly, including 6 monthly download of CPAP usage, and were counselled to encourage continued nightly CPAP use. At study conclusion a PSG (on CPAP) with before and after sleep retinal photographs was obtained.

Note: The study described in this manuscript has minor deviations from the registered clinical trial (ACTRN12620000694910). In particular, patients aged ≥43yrs were recruited, smokers were not excluded but smoking status was noted, and the RFM/CPAP sub-group included patients with an RDI ≥ 29 events/hr.

### Data analysis

All statistical analyses were performed using SPSS version 24.0 (IBM SPSS Statistics for Windows, Version 24.0. Armonk, NY: IBM Corp.).

Main and RFM/CPAP Sub-Group data were expressed as mean (± standard deviation) or median (interquartile range) for continuous variables, and frequency and percentage for categorical variables. Spearman’s rank-order correlation were used to test for relationships between continuous variables. Differences between the Main Group and RFM/CPAP sub-group were tested using independent sample t-tests or Mann-Whitney U tests for continuous variables, and Chi-Square tests for categorical variables. A paired t-test was used to examine overnight change (morning minus evening) in CRAE and CRVE (ΔCRAE; ΔCRVE, respectively).

Backwards, stepwise, multiple linear regression (variables removed at p>0.1) was used to establish predictive variables for retinal vessel size and overnight change using a strategy of first establishing non-SDB related predictive factors operating in the data set (Base Models) and then assessing the impact of adding individual SDB-variables to the base models (SDB Variable Models).

Non-SDB variable predictor candidates were identified in two ways: 1) from published models for retinal vessel size [18]; and 2) univariate associations (at p<0.05) within the study data set. A ‘study group’ factor (Main Group v RFM Sub-Group) was included to determine whether the same set of variables were operating in both groups. Collinear variables were identified by collinearity tests–variance inflation factor (VIF>10), condition index (>30) and variance proportions (>0.5)–and were removed based on weaker univariate associations with the dependent variable.

SDB variables were skewed (Kolmogorov-Smirnov Test for Normality) and, therefore, log transformed (e.g. ln AHI+1) before being individually added to the base models as required. Any improvement in the model R^2^ values (ΔR^2^), was used to determine whether adding the SDB variable improved overall model performance. Diagnostic residual plots (including residuals versus fitted values and normal probability plots of the residuals) were used to check the adequacy of the fitted models.

For the RFM/CPAP intervention study, paired t-tests were used to identify differences in retinal vessel diameter size (ie evening CRAE, CRVE and overnight change in CRAE/CRVE) over 12 months. Spearman’s Rho rank correlation was used to test for correlations between the change (Δ) in retinal vessel diameter, CPAP use, Δ base model variables (age, systolic and diastolic blood pressure) and SDB variables. Multiple linear regression analysis was used to establish predictive variables for: 1) Δ evening retinal vessel diameter, and 2) Δ overnight change in retinal vessel diameter.

## Results

### Cross-sectional study–main group

This section describes results for the primary study analyses which focused on determining cross-sectional associations for non-SDB (Base Models) and SDB severity metrics (SDB Variable Models) with measured evening CRAE, CRVE, and overnight change in CRAE and CRVE, in Main Group participants (n = 264).

### Descriptive data—main group

Table 1 shows Main Group anthropometric, cardiovascular risk, SDB and retinal vessel metrics.

**Table 1:** Main group demographics and sleep disordered breathing status. Demographics and SDB status of the Main Group at baseline, presented as mean ± SD, median (IQR) frequency, or n (%), where appropriate, plus range.

	**N**	**Mean ± S.D**	**Range**
**Anthropometrics**			
Age (years)	264	58.5 ± 8.9	43–79
Height (cm)	263	167.0 ± 9.6	144.0–191.5
Weight (kg)	263	88.8 ± 21.0	41.6–186.3
BMI (kg/m^2^)	263	31.8 ± 7.0	17.1–64.5
Neck circumference (cm)	261	39.7 ± 4.6	30.0–56.5
Waist circumference (cm)	261	104.6 ± 15.1	50.0–162.0
Hip Circumference (cm)	261	111.3 ± 14.3	69.0–179.0
WHR (a.u.)	261	0.94 ± 0.08	0.52–1.14
Systolic BP (mmHg)	262	128.5 ± 15.1	94–176
Diastolic BP (mmHg)	262	73.2 ± 11.5	42–109
MAP BP (mmHg)	262	94.6 ± 9.8	67–128
	**N**	**Frequency**	**Percentage**
Gender (male)	264	141	53.4
Ethnicity (Caucasians)	264	170	64.4
Hypertension History	264	114	43.2
Hypercholoesterolemia History	264	117	44.3
Smoking History (including 6 current smokers)	264	118	44.7
**Framingham Risk Level**			
FRS risk level	**Male**	**Female**	**Total**
	**[n (%)]**		
Low	41 (31.5%)	74 (63.2%)	115 (46.6%)
Intermediate	50 (38.5%)	37 (31.6%)	87 (35.2%)
High	39 (30.0%)	6 (5.1%)	45 (18.2%)
**Blood Test Results**			
	**N**	**Mean** ± **S.D**	**Range**
Total cholesterol (mmol/l)	259	5.1 ± 1.0	3.0–7.9
Triglycerides (mmol/l)	258	1.5 ± 0.7	0.5–4.9
HDL (mmol/l)	250	1.3 ± 0.3	0.7–2.7
LDL (mmol/l)	250	3.1 ± 0.9	1.2–6.0
Blood glucose level (fasting, mmol/l)	264	5.4 ± 0.6	3.8–6.9
**Sleep Study Parameters**			
	**N**	**Median (IQ Range)**	**Range**
AHI (events/hr)	264	12.7 (4.4–26.5)	0–103.9
RDI (events/hr)	264	24.7 (14.2–42.4)	1.1–104.2
AI (events/hr)	264	27.6 (19.5–40.1)	5.9–100.5
ODI ≥3% (events/hr)	264	4.4 (1.2–10.3)	0–73.0
SaO2 <90% (% TST)	264	0.6 (0–3.3)	0–98.2
**Retinal Vessel Diameter Parameters**			
	**N**	**Mean ± S.D**	**Range**
Evening CRAE (μm)	256	143.32 ± 12.99	98.65–181.86
Evening CRVE (μm)	255	213.70 ± 18.94	150.88–272.46
Evening AVR (a.u)	256	0.67 ± 0.05	0.55–0.84
Morning CRAE (μm)	252	143.81 ± 13.77	103.16–180.76
Morning CRVE (μm)	252	215.25 ± 20.55	150.20–278.13
Morning AVR (a.u)	252	0.67 ± 0.05	0.53–0.89

SD = Standard deviation; IQ range = 25–75 interquartile range; BMI = Body Mass Index; WHR = waist/hip ratio; BP = blood pressure; MAP = Mean Arterial Pressure; HDL = high-density lipoprotein; LDL = low-density lipoprotein; AHI = Apnea-Hypopnea Index; RDI = Respiratory Disturbance Index; AI = Arousal Index; ODI = Oxygen Desaturation Index; SaO2 = Oxygen Saturation; CRAE = Central *retinal* arteriolar equivalent; CRVE = central *retinal* venular equivalent; AVR = arterio-venule-ratio; TST = total sleep time.

### Univariate analyses -main group

*Evening CRAE and CRVE*. Cross-sectionally, both CRAE and CRVE correlated negatively with age, while CRAE corelated negatively with a number of body size metrics, systolic BP and Framingham risk level (all R>-0.13, p<0.05). CRVE values correlated negatively with systolic BP and positively with diastolic BP. There were no correlations (all p>0.05) between any SDB variable and CRAE, CRVE or AVR (S1 Table).

*Overnight Change in CRAE and CRVE*. There was no overnight change in mean CRAE, however, mean CRVE increased slightly overnight by an average 1.73μm (S.D 8.99 μm, p = 0.003). For CRAE overnight change, but not CRVE, there was a negative correlation (R>-0.19, P<0.01) with systolic and diastolic BP change (S1 Table) i.e. the difference in overnight CRAE (Morning-Evening) decreased as the difference in overnight BP increased. Table 2 shows evening, morning and overnight change data for CRAE, CRVE and AVR grouped by AHI category.

**Table 2:** Main group data for evening, morning and overnight change in CRAE and CRVE and AVR grouped by AHI category.

	AHI <5 events/hr (n = 70)	AHI 5–15 events/hr (n = 75)	AHI 15–30 events/hr (n = 51)	AHI > 30 events/hr (n = 52)
**Evening CRAE (μm)**	145.1 ± 12.3	144.0 ± 13.7	139.2 ± 13.0	145.1 ± 12.7
**Morning CRAE (μm)**	146.3 ± 13.2	145.0 ± 13.2	138.0 ± 14.1	145.0 ± 13.8
**Evening CRVE (μm)**	216.8 ± 18.2	212.5 ± 20.7	208.8 ± 19.0	217.3 ± 17.3
**Morning CRVE (μm)**	219.6 ± 21.0	214.3 ± 20.5	210.2 ± 20.9	217. 8 ± 18.3
**Evening AVR (a.u)**	0.67 ± 0.06	0.68 ± 0.05	0.67 ± 0.05	0.67 ± 0.05
**Morning AVR (a.u)**	0.67 ± 0.06	0.68 ± 0.05	0.66 ± 0.06	0.67 ± 0.05
**Delta CRAE (μm)**	1.18 ± 6.40	0.98 ± 5.50	-1.20 ± 5.25	-0.09 ± 5.50
**Delta CRVE (μm)**	2.81 ± 8.51	1.82 ± 9.91	1.33 ± 8.58	0.53 ± 8.70

Data are mean ± SD.

CRAE = Central retinal arteriolar equivalent; CRVE = central retinal venular equivalent; AVR = arterio-venule-ratio; AHI = apnoea-hypopnea index; Delta = overnight change.

### Multiple linear regression models -main group

*Base Models*. Evening CRAE. The final model (S3A Table) explained 18% of the variance in CRAE (R^2^ = 0.178, p < 0.001). Systolic BP was the most significant predictor (p < 0.001) with a 0.18μm decrease in CRAE per mmHg BP increase. Other predictors (p<0.05) were age (2.47μm decrease per decade), ethnicity (4.45 μm smaller in Caucasians versus Non-Caucasians) and height (0.24 μm decrease per cm increase). A positive smoking history was associated with ~3.08 μm increase in CRAE. Pupil dilation and study group were not considered to be predictors both p>0.05.

Evening CRVE. The final model (S3B Table) explained 25% of the variance in CRVE (R^2^ = 0.252, p < 0.001). CRVE values tended to decrease with age, however,.Caucasian ethnicity was the most prominent predictor (p<0.001) with an 11.52 μm decrease in CRVE if Caucasian versus Non-Caucasian. Diastolic BP (0.34 μm increase in CRVE per mm Hg BP increase), hypertension history (6.5 μm decrease) and smoking history (4.6 μm increase) were the only other predictors (p<0.05) in the final base model. PM dilation and study group were not considered to bepredictors (both p>0.05).

Overnight Change in CRAE/CRVE. When the variables from the above CRAE and CRVE final base models were entered into a multiple linear regression analysis for effects on overnight change in retinal vessel diameters, the models all failed (p>0.1), i.e., no model explained the variance in overnight change for CRAE or CRVE.

*SDB Variable Models*. *Evening CRAE and CRVE*. No SDB variable or category emerged as a predictor (all p>0.05) for either evening CRAE or CRVE and their inclusion resulted in little improvement in the strength of model predictions (CRAE: ΔR^2^ ranged from 0.000 to 0.014, S4A Table; CRVE: ΔR^2^ ranged from 0.000 to 0.008, S4B Table).

Overnight Change in CRAE/CRVE. Adding SDB variables to the base models did not enhance the ability of these models to predict overnight change in CRAE or CRVE (all p > 0.1).

### Cross-sectional study—RFM/CPAP sub-group

This section describes comparison of descriptive data for the RFM/CPAP Sub-Group (n = 85) versus those Main Group partcipants who were not recruited into the intervention study (n = 179), together with cross-sectional associations for non-SDB (Base Models) and SDB severity metrics (SDB Variable Models) with measured evening CRAE, CRVE, and the overnight change in CRAE/CRVE, in the RFM/CPAP Sub-Group participants.

### Descriptive data and univariate analysis-RFM/CPAP sub-group

RFM/CPAP Sub-group participants differed from the Main Group participants who were not recuited into the sub-group (all p < 0.04) for gender composition, most measures of body size, hypertension history, overall cardiovascular risk, and all SDB variables. Thus, the applied selection criteria enriched the RFM/CPAP-Sub Group with more males and more severe SDB. However, there were no differences for retinal vessel diameter metrics between the RFM/CPAP Sub-Group and those Main Group participants who were not recruited into the sub-group (all p>0.06), including overnight change for both CRAE and CRVE (S2A & S2B Table).

Correlation analyses were not performed (see below).

### Multiple linear regression models–RFM/CPAP sub- group

*Base Models*. The study group variable (Main Group versus RFM/CPAP Sub-Group) did not reach p<0.05 for any of the cross-sectional Base Models. Consequently, univariate analyses (correlations) were not performed and the Base Models for the RFM/CPAP Sub-Group were constructed using the same variables as emerged in the Main Group Base Model analysis.

Evening CRAE. For evening CRAE, the base model explained 20% of the variance (R^2^ = 0.20, p<0.02), with Caucasian ethnicity being the only contributingvariable (B = -9.167, p<0.01).

Evening CRVE. For evening CRVE, the base model explained 31% of the variance (R^2^ = 0.309, p<0.001), with Caucasian ethnicity (B = -15.938) and diastolic BP (B = 0.517) being predictors (both p<0.02).

Overnight Change in CRAE/CRVE. For overnight change in CRAE, the base model explained only 14% of the variance (R^2^ = 0.144, p < 0.01), with overnight change in diastolic BP being the only contributing variable (B = -0.211, p < 0.01). While for overnight change in CRVE, the base model again only explained only 20% of the variance (R^2^ = 0.200, p <0.01), with age and overnight change in diastolic BP both emerging as predictors (both p < 0.01).

*SDB Variable Models*. There was a interaction (p<0.05) effect on CRAE for the [SDB variable by study group ie Main Group vs RFM/CPAP Sub-Group]. Therefore, SDB variable testing was undertaken in the RFM/CPAP Sub-Group.

Evening CRAE. For CRAE, there were effects found for the [AHI by study group] (p = 0.038) and [AHI category by study group] (p = 0.018) interactions indicating there was a group effect operating and the RFM/CPAP Sub-Group required separate investigation. When added individually to the Base Model in the RFM/CPAP Sub-Group, Ln (AHI + 1) and AHI severity category (AHI > 30 events/hr) had an effect on CRAE (p<0.05). When Ln(AHI+1) was added to the model it explained 25% of the variance (R^2^ = 0.253, p<0.03, ΔR^2^ = 0.053, S5 Table) with a per-unit increase ln(AHI+1) = 1 associated with a 0.046μm increase in CRAE. Similarly, when AHI severity category was added to the model it explained 27% of the variance (R^2^ = 0.270, p<0.012, ΔR^2^ = 0.070).

Evening CRVE and Overnight Change in CRAE/CRVE. None of the [SDB variable by study group] interactions had an effect (all p > 0.1). Consequently, no SDB variable testing was undertaken in the RFM/CPAP Sub-Group for CRVE or for overnight change in CRAE or CRVE.

### RFM/CPAP sub-group intervention study

This section describes results for the 12 month RFM/CPAP interventional study (n = 66) which aimed to evaluate whether stabilising known cardiovascular disease risk factors (hypertension, hypercholesterolemia), while concurrently treating OSA with continuous positive airway pressure (CPAP), would result in alterations in evening CRAE, CRVE and overnight change in CRAE and CRVE.

### CPAP usage—RFM/CPAP intervention study

Over the intervention period, RFM/CPAP Sub-Group (n = 65) CPAP compliance averaged 5.2 ± 1.8 hrs per night (mean±SD; range: 0.5 to 8.0 hrs) with an average 69.8 ± 24.6% of nights with compliance >4 hours (range: 3 to 99 days).

*Univariate Analyses*. *Evening CRAE*. There was no difference in group mean evening CRAE value across the 12 month RFM/CPAP period and no correlations between individual changes in evening CRAE and baseline age, change in evening systolic and diastolic PM, all baseline SDB variables, and average CPAP hours used (all p > 0.1).

**Fig 2:**
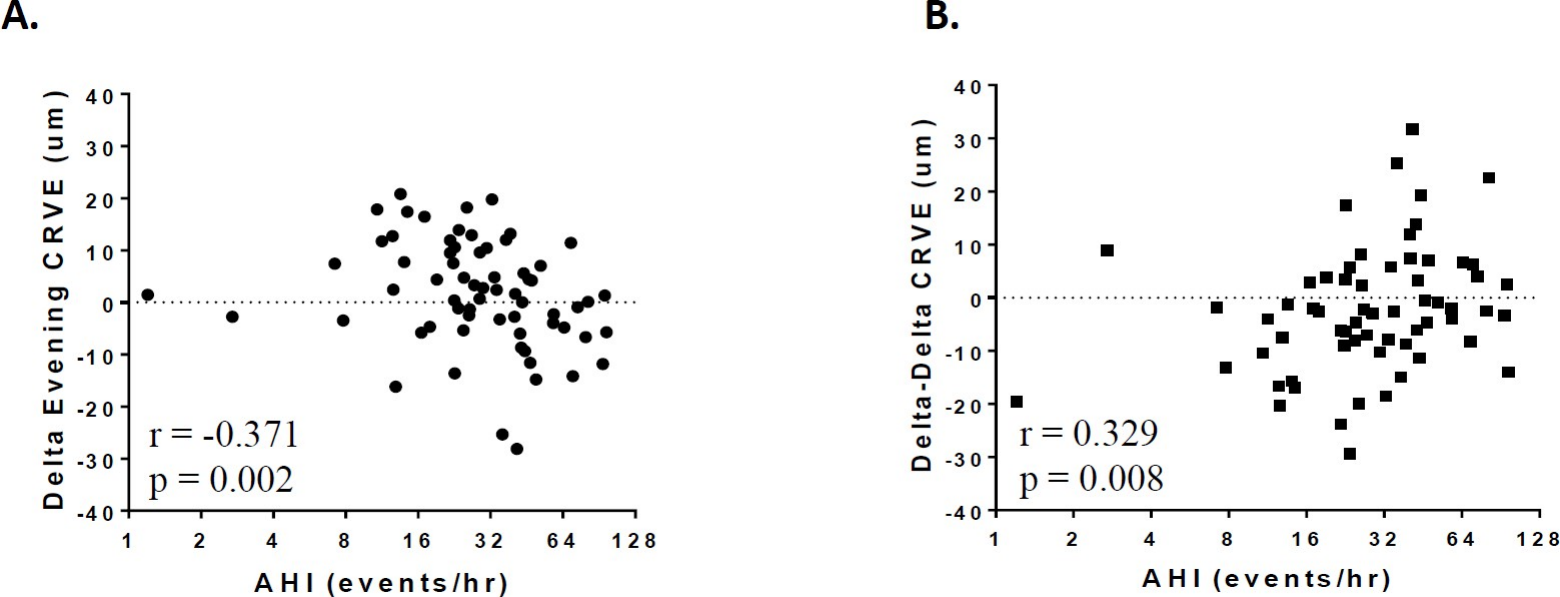
CRVE change with 12 months of RFM/CPAP treatment. Semi-log scatterplots showing change in evening CRVE (A) and change in the overnight difference (morning–evening) for CRVE (Delta-Delta CRVE) (B) plotted against starting AHI across 12 months of RFM/CPAP treatment. Each point represents an individual subject’s data. Delta Evening CRVE represents the change in evening CRVE over the 12 months (12 months−baseline); Delta-Delta CRVE represents the overnight change in CRVE over the 12 months ([morning–evening] _12 months_−[morning–evening] _baseline_)_._ r = Spearman’s Correlation Coefficient.

Evening CRVE. There was no difference in group mean evening CRVE value across the 12 month RFM/CPAP period (p = 0.2) and no s correlations between individual changes in evening CRVE and baseline age, change in systolic and diastolic evening BP, and average CPAP hours used (all p>0.05). However, there were negative correlations with starting AHI (r = -0.371, p < 0.01), RDI (r = -0.348, p < 0.01) and ODI > 3% (r = -0.303, p < 0.02) but not AI or SaO2<90% (both p > 0.1). Fig 2A shows change in evening CRVE value across over the 12 month RFM/CPAP period plotted against starting AHI value.

Overnight Change in CRAE and CRVE. There were no differences in group mean overnight change in CRAE or CRVE over the 12 month RFM/CPAP period (both p > 0.09) and no correlations between overnight individual changes in CRAE and CRVE and baseline age, change in systolic and diastolic evening BP, average CPAP hours used and all SDB variables (all p>0.5). However, there was a positive correlation between baseline AHI and overnight change in CRVE over 12 months of CPAP (r = 0.329, p = 0.01). Fig 2B shows the difference in the overnight change in CRVE over 12 months of RFM/CPAP treatment plotted against baseline AHI.

### Multiple linear regression models—RFM/CPAP sub-group intervention study

*Change in Evening CRAE and Change in Overnight CRAE Difference across RFM/CPAP Period*. No predictors (all P>0.1) emerged from multiple regression models examining change in evening CRAE or change in overnight CRAE differences across the 12 month intervention.

*Change in evening CRVE*. Across the 12 month RFM/CPAP intervention, there was a 6.13 μm (SE: 2.56 μm) reduction in evening CRVE values for individuals with an AHI >30 events/hr (p = 0.02, R^2^ = 0.13).

*Change in Overnight CRVE Difference across RFM/CPAP period*. Across the 12 month RFM/CPAP intervention, there was a 7.05 μm. (SE: 2.98 μm) increase in the overnight difference for CRVE values for individuals with an AHI >30 events/hr (p = 0.02, R^2^ = 0.18).

## Discussion

The primary focus of the present study was to test for relationships, in a non-diabetic sleep-clinic patient cohort, between polysomnography determined SDB severity and retinal micro-vasculature morphology. Overall no such relationships were detected, but when a subgroup enriched with more severe levels of SDB was examined, a greater AHI value was associated with a larger evening-measured CRAE value. However, the effect size for this relationship was small, with only a ~2.5 μm (~1.7%) increase in CRAE over the AHI range 5 to 30 events/hr.

Our study is unique in that we tested for acute overnight effects of SDB on retinal vascular morphology. While there was no group mean overnight change in CRAE, mean CRVE values measured in the morning, immediately upon awakening, were on average slightly larger than the prior evening values. Multivariable models failed to identify predictors for this outcome, except in a sub-group characterised by more severe SDB, where older individuals had less of an overnight change in CRVE. Another feature of our experimental design was a 12 month monitored RFM/CPAP intervention phase characterised by relatively high rates of compliance. In the subgroup enrolled in this part of the study, across the RFM/CPAP period, there were no detected group mean changes in CRAE or CRVE evening values. However, individuals with more severe SDB had a decreased evening CRVE and, correspondingly, an increased overnight change in CRVE after 12 months of RFM/CPAP treatment.

In addition, this is the first study to demonstrate an effect of sleep, or circadian variation, on retinal vascular morphology. There is a small difference in morning CRVE compared to the evening CRVE, and this change increases in those with more severe SDB after treatment. Circadian changes in the retina have long been recognised [19]. The timing of retinal vasculature assessments should be considered in future studies.

In this study any identified impacts of SDB severity on retinal vascular morphology were all characterised by effect sizes that were relatively small. Therefore, in this non-diabetic cohort, where other cardiovascular disease risk factors were minimised/controlled, it seems unlikely that such associations reflect a major pathophysiological interaction between SDB and retinal microvascular morphology.

### Study cohort characteristics

In interpreting our results it is important to understand the characteristics of the study cohort. This was not a randomised community-recruited population-based study. We targeted a specific clinic group (i.e. patients referred to a sleep clinic for assessment of SDB) and then screened for and eliminated diabetes as a cardio-vascular risk factor. Consequently, our study group was also not a generalised sleep clinic population sample. Rather, we aimed to test for associations between specific SDB metrics and specific micro-vascular characteristics (retinal vessel calibre) in an environment where the influence of known cardio-vascular disease risk factors was absent, minimised or controlled. The rationale for this approach assumed that SDB influences were likely to be small compared with well-known systemic risk factors, such as diabetes, and therefore, would be more likely detected in the absence of such major confounders. Previous published studies that have attempted to quantify associations between retinal vessel calibre and SDB have not attempted to minimise other cardiovascular risk factors [11,12,20].

### Evening CRAE

Group mean values for evening CRAE in our Main Group were ~12% smaller than reported for the younger Inter99 Eye study non-sleep cohort [21,22], but were ~27–29% smaller than reported for similar aged cohorts in the Blue Mountains [23] and Beaver Dam community population studies [18].

In our multivariate Base Models, Main Group evening CRAE values were predicted by a combination of “fixed at the time of study” individual characteristics (age, ethnicity, height, smoking history) and one “physiological variable” (systolic BP; see S3A Table). These outcomes are consistent with previously published analyses involving larger community-based cohorts [21,22,24,25].

It should be emphasised that our Base Model for evening CRAE explains only ~18% of the total variance, leaving the vast majority of the between subject variance unexplained. This is not unusual, with published multivariate models developed using larger cohorts explaining only 8.2 to 31.7% of the variance [24,26–28].

In the Main Group, no SDB variable correlated with CRAE, adding SDB variables to the Base Model did not improve the predictive power of the model and no SDB variable emerged as a predictor. However, in the more severe SDB RFM/CPAP Group, when AHI and AHI category were added to the Base Model, the model now explained ~25% of the variance and both AHI and AHI category emerged as predictors for a slightly larger evening CRAE. This may be a consequence of hypoxia, as AHI is a surrogate for intermittent hypoxia, and chronic hypoxia has been demonstrated in animal models to result in retinal angiogenesis [29]. However, the effect size is small (see S5 Table) and in the opposite direction to the findings of Tong et al [12]. Unlike this study [12], our retinal vascular measurements were contemporaneous with the sleep study.

A unique feature of the present study is the measurement of both evening and morning retinal vessel diameters across a night where sleep was monitored and SDB quantitatively assessed. Multivariate models identified change in diastolic BP as a negative predictor but no SDB variable was identified as a predictor for overnight change in CRAE.

### Evening CRVE

Group mean values for evening CRVE in our Main Group were ~15% smaller than reported for the younger Inter99 Eye study community cohort [21,22], but were also ~5–7% smaller than reported for similar aged cohorts in the Blue Mountains [23] and Beaver Dam community population studies [18].

In multivariate Base Models, Main Group evening CRVE values were predicted by a combination of “fixed at the time of study” individual characteristics (ethnicity, smoking history, hypertension history; with age and height borderline) and one “physiological variable” (diastolic BP, with systolic BP borderline; see S3B Table). These outcomes are similar to our findings for CRAE and consistent with previously published analyses involving larger community-based cohorts [21,23]. The positive association between diastolic BP and evening CRVE values is in contrast with the negative association recently reported by Dervenis and co-workers [30].

As for CRAE, it should be emphasised, that our Base Model explains only ~25% of the total variance in Main Group evening CRVE, leaving the vast majority of the between subject variance unexplained. Again, this is not unusual, with published models developed using larger cohorts explaining only 4.2 to 21% of the variance [24,26–28].

In the Main Group, no SDB variable correlated with CRVE values and when SDB variables were added to the base model there were no improvements in the amount of variance explained and no SDB variable emerged as a predictor. This outcome differs from that of Shankar et al [11] who proposed that after adjusting for age, gender, BMI, hypertension, diabetes and lipid levels, increasing AHI was associated with a wider CRVE. However, these authors studied a diabetic population with high LDL and HDL levels, in contrast to the present study where lipid levels were well controlled and diabetes was excluded. Previous authors have suggested that relationships between SDB and retinal venular widening may be associated with concomitant factors such as inflammation, metabolic abnormalities and dysglycemia [31], factors that were absent or controlled in the present study.

For CRVE in the Main Group, group mean values were slightly larger in the morning versus evening (see Table 1). However, the Base Model failed to explain any of the associated variance. In the RFM/CPAP Sub-Group mean CRVE values were ~ 0.5% greater in the morning versus evening and there was a negative correlation between overnight change in CRVE and overnight change in both systolic and diastolic BP. For this sub-group, the Base Model only explained ~18% of the variance in overnight change in CRVE, with age as a negative predictor ie older subjects had a smaller overnight change. No SDB variable predicted overnight change in CRVE, in either the Main or CPAP Sub-Group. These findings suggest that while overnight widening of CRVE may be related to overnight changes in blood pressure, this effect appears to be modified by age.

### RFM/CPAP intervention study

When a sub-group with a wide range of SDB severity were individually stabilised on medical therapy, monitored and given CPAP therapy over a 12 month period, there was no change in group mean evening CRAE or CRVE values or the overnight change in CRAE or CRVE. However, evening CRVE values tended to decrease over the CPAP period, especially for those with more severe SDB (AHI>30 events/hr; see Fig 2A), while overnight change in CRVE for these individuals tended to increase (see Fig 2B), primarily because of the fall in evening CRVE values. This decrease in evening CRVE over the 12 month RFM/CPAP period, however, was not related to a change in blood pressure or the number of hours of CPAP used. While the mechanism remains uncertain, a reduction in CRVE values (particularly for individuals with larger reductions of 10–30 μm, see Fig 2A) is tending in the direction associated with reduced risk for both stroke [32] and ischemic heart disease (IHD), since a wider CRVE has also been reported to carry increased risk for IHD [21].

In summary, treatment of OSA with CPAP (combined with medical treatment to control lipid levels and blood pressure) had some effect over 12 months in reducing evening CRVE, and thus perhaps reducing stroke risk. However, this change was not dependent on the amount of CPAP used, and therefore may reflect the impact of all the medical interventions aimed at reducing/stabilising vascular risk factors, rather than the elimination of SDB.

### Study limitations

The study has a number of strengths and limitations. Firstly, it is essentially descriptive and exploratory in nature, consequently, its results should be interpreted accordingly. In addition, given the number of relationships explored, p-values provided should be regarded as only a guide to any potential relationships.

Inclusion/exclusion criteria eliminated a major cardiovascular risk factor, diabetes, and controlled for others, eg treatment of hypertension and hypercholesterolaemia, but allowed for major anthropometric influences, e.g. age and body size. This approach aimed to avoid effects of known cardio-vascular disease confounders that typically occur in large community recruited cohorts, while simultaneously avoiding the limitations of very small groups, often produced by protocols that attempt to eliminate all known risk factors. In addition, the majority of the subjects had retinal microvasculature diameters that were within ranges seen in healthy subjects. The failure to identify any major change following treatment may be a consequence of a floor effect, where vessels are unable to change any further. Despite our efforts, classic risk factors were still present in the group and in the process, we have also restricted our ability to examine interactions between SDB and retinal microvascular anatomy when uncontrolled classic risk factors such as diabetes are fully present. The lack of a demonstrated association between SDB metrics and retinal vessel diameters needs to be interpreted with this latter point in mind. It is possible that SDB may have had an additive or even multiplicative effect on uncontrolled classic cardiovascular risk factors. However, such an interaction would have been missed by our current study design.

## Conclusion

In this study of non-diabetic patients presenting to a sleep clinic for investigation of SDB, total variance in retinal vessel size was partially explained by a combination of anthropometric characteristics and known predictors, with measures of SDB providing very little to no contribution. In a more severely affected sub-group, increased SDB severity was a minor predictor for increased evening CRAE values, while 12 months of RFM/CPAP treatment was associated with a small decrease in evening CRVE values, which may reflect modifiable cardiovascular disease risk, particularly for patients with more severe levels of SDB. However, overall findings in this non-diabetic sleep clinic cohort do not support a major impact of SDB on the morphology of the retinal microvasculature, suggesting that SDB may also have fairly minimal impact on the cerebral microvasculature, particularly when classic cardiovascular risk factors are absent or well controlled.

## Supporting information

S1 Checklist (PDF)

S1 Table Univariate correlations- main group (n = 264). Spearman’s Rank Correlation Coefficients for associations between anthropometric/demographic characteristics, Framingham Risk, blood test results, and SDB variables versus retinal vessel diameters and their overnight change—Main Group.
(DOCX)

S2 Table RFM/CPAP sub-group versus main group (minus RFM/CPAP sub-group) participant characteristics. Continuous A) and categorical B) data comparisons for RFM/CPAP Sub-Group participants (n = 85) versus Main Group minus CPAP Sub-Group participants (n = 179).
(DOCX)

S3 Table Multiple linear regression models for evening retinal vessel diameters using non -SDB variables (base models)—main group (n = 264). Base Models showing non- SDB variable predictors for evening CRAE (A) and evening CRVE (B) for the Main Group.
(DOCX)

S4 Table Multiple linear regression models for evening retinal vessel diameters using SDB variables (SDB models)—main group (n = 264). Results of adding each SDB variable individually to the Base Model for evening CRAE (A) and evening CRVE (B) in the Main Group.
(DOCX)

S5 Table Multiple linear regression models for evening CRAE using SDB variables (SDB models)- RFM/CPAP sub-group (n = 85). Results of adding LnAHI+1 and AHI Category into the Base Model for CRAE in the RFM/CPAP Sub-Group.
(DOCX)

S1 File Compressed file archive–SPSS data/anonymysed data. (ZIP)

S2 File Compressed file archive–SPSS syntax. (ZIP)

S3 File Compressed file archive: Anonymised raw data/other. (ZIP)

S4 File (ZIP)

S1 Study protocol (PDF)
