# Supplementary material for: Sleep disordered breathing has minimal association with retinal microvascular diameters in a non-diabetic sleep clinic cohort
Source: PLoS One. 2023 Jan 10;18(1):e0279306. doi: 10.1371/journal.pone.0279306 (PMC9831323; doi:10.1371/journal.pone.0279306)
Supplement: S1 Table — Spearman’s Rank Correlation Coefficients for associations between anthropometric/demographic characteristics, Framingham Risk, blood test results, and SDB variables versus retinal vessel diameters and their overnight change—Main Group. (DOCX) [file pone.0279306.s002.docx]

**Table S1: Univariate Correlations- Main Group (n=264)**

Spearman’s Rank Correlation Coefficients for associations between anthropometric/demographic characteristics, Framingham Risk, blood test results, and SDB variables versus retinal vessel diameters and their overnight change - Main Group.

|  | **Evening CRAE (μm)** | | **Evening CRVE (μm)** | | **Evening AVR (a.u)** | |
| --- | --- | --- | --- | --- | --- | --- |
|  | **R** | **p** | **R** | **p** | **R** | **p** |
| **Anthropometric/Demographic Characteristics** | | | | | | |
| Age (years) | -0.236 | < 0.001 ^+^ | -0.255 | < 0.001 ^+^ | 0.064 | 0.307 |
| Height (cm) | -0.174 | 0.005 ^+^ | -0.134 | 0.033 ^+^ | 0.019 | 0.763 |
| Weight (kg) | -0.166 | 0.008 ^+^ | -0.092 | 0.143 | -0.096 | 0.126 |
| BMI (kg/m^2^) | -0.109 | 0.082 | -0.024 | 0.699 | -0.129 | 0.039 ^+^ |
| Neck circumference (cm) | -0.161 | 0.010 ^+^ | -0.065 | 0.300 | -0.096 | 0.126 |
| Waist circumference (cm) | -0.209 | 0.001 ^+^ | -0.077 | 0.221 | -0.180 | 0.004 ^+^ |
| Hip Circumference (cm) | -0.139 | 0.027 ^+^ | -0.045 | 0.479 | -0.144 | 0.021 ^+^ |
| WHR (a.u.) | -0.180 | 0.004 ^+^ | -0.079 | 0.208 | -0.115 | 0.067 |
| Systolic BP (mmHg) | -0.174 | 0.005 ^+^ | -0.133 | 0.035 ^+^ | -0.068 | 0.276 |
| Diastolic BP (mmHg) | 0.094 | 0.136 | 0.188 | 0.003 ^+^ | -0.159 | 0.012 ^+^ |
| MAP BP (bpm) | -0.134 | 0.032 ^+^ | -0.039 | 0.535 | -0.132 | 0.035 ^+^ |
| **Framingham Risk Score** | | | | | | |
| Framingham risk level (a.u.) | -0.238 | < 0.001 ^+^ | -0.210 | 0.001 ^+^ | -0.036 | 0.582 |
| **Blood Test Results** | | | | | | |
| Total cholesterol (mmol/l) | 0.095 | 0.135 | 0.107 | 0.090 | -0.010 | 0.878 |
| Triglyceride (mmol/l) | -0.018 | 0.774 | -0.004 | 0.949 | -0.022 | 0.727 |
| HDL (mmol/l) | 0.130 | 0.043^+^ | 0.064 | 0.321 | 0.084 | 0.193 |
| LDL (mmol/l) | 0.055 | 0.395 | 0.106 | 0.100 | -0.059 | 0.357 |
| Blood glucose level (fasting, mmol) | 0.033 | 0.595 | 0.050 | 0.424 | -0.019 | 0.765 |
| **SDB Variables** | | | | | | |
| AHI (events/hr) | -0.069 | 0.273 | -0.064 | 0.311 | 0.004 | 0.955 |
| RDI (events/hr) | -0.107 | 0.087 | -0.021 | 0.743 | -0.067 | 0.288 |
| AI (events/hr) | -0.046 | 0.464 | 0.015 | 0.812 | -0.022 | 0.727 |
| ODI >3% (events/hr) | -0.043 | 0.497 | -0.010 | 0.875 | -0.025 | 0.691 |
| SaO2 <90% (% TST) | -0.079 | 0.206 | -0.065 | 0.300 | -0.016 | 0.796 |
| **Overnight Change Retinal Vessel Size** | | | | | | |
|  | **Overnight CRAE (μm)** | | **Overnight CRVE (μm)** | | **Overnight AVR (a.u)** | |
| Overnight systolic BP (mmHg) | -0.202 | 0.001 ^+^ | -0.045 | 0.484 | -0.125 | 0.050 ^+^ |
| Overnight diastolic BP (mmHg) | -0.198 | 0.002 ^+^ | -0.086 | 0.180 | -0.099 | 0.123 |

^+^ Indicates significant (p<0.05) correlations.

SDB=Sleep Disordered Breathing, R = Spearman’s Rank Correlation Coefficient; BMI = Body Mass Index; WHR = waist/hip ratio; BP = blood pressure; MAP = Mean Arterial Pressure; HDL = High-Density Lipoprotein; LDL = Low-Density Lipoprotein; AHI = Apnea-Hypopnea Index; RDI = Respiratory Disturbance Index; AI = Arousal Index; ODI = Oxygen Desaturation Index; SaO2 = Oxygen Saturation; CRAE = Central Retinal Arteriolar Equivalent; CRVE = Central Retinal Venular Equivalent; AVR = arterio-venule-ratio; TST = total sleep time.
