# Supplementary material for: Sleep disordered breathing has minimal association with retinal microvascular diameters in a non-diabetic sleep clinic cohort
Source: PLoS One. 2023 Jan 10;18(1):e0279306. doi: 10.1371/journal.pone.0279306 (PMC9831323; doi:10.1371/journal.pone.0279306)
Supplement: S2 Table — Continuous A) and categorical B) data comparisons for RFM/CPAP Sub-Group participants (n = 85) versus Main Group minus CPAP Sub-Group participants (n = 179). (DOCX) [file pone.0279306.s003.docx]

**Table S2: RFM/CPAP Sub-Group versus Main Group (minus RFM/CPAP Sub-Group) Participant Characteristics**

Continuous A) and categorical B) data comparisons for RFM/CPAP Sub-Group participants (n=85) versus Main Group minus CPAP Sub-Group participants (n=179).

**A)**

| **Variable** | **Main Group-RFM/CPAP Sub-Group** | **RFM/CPAP Sub-Group** | **p** |
| --- | --- | --- | --- |
|  | **[mean ± SD / median (IQR)]** | |  |
| **Anthropometrics** | | | |
| Age | 57.9 ± 8.5 | 59.8 ± 9.6 | 0.112 |
| Height (cm) | 166.7 ± 9.3 | 167.8 ± 10.2 | 0.384 |
| Weight (kg) | 85.8 ± 19.2 | 95.0 ± 23.3 | 0.002^+^ |
| BMI (kg/m^2^) | 30.9 ± 6.4 | 33.8 ± 7.8 | 0.002^+^ |
| Neck circumference (cm) | 38.9 ± 4.2 | 41.5 ± 4.8 | < 0.001^+^ |
| Waist circumference (cm) | 102.0 ± 13.6 | 110.1 ± 16.8 | < 0.001^+^ |
| Hip Circumference (cm) | 109.7 ± 13.0 | 114.8 ± 16.1 | 0.006^+^ |
| WHR (a.u) | 0.93 ± 0.08 | 0.96 ± 0.09 | 0.011^+^ |
| Systolic BP (mmHg) | 127.4 ± 15.6 | 131.0 ± 13.8 | 0.067 |
| Diastolic BP (mmHg) | 72.7 ± 11.6 | 74.2 ± 11.3 | 0.322 |
| MAP BP (mmHg) | 94.0 ± 9.8 | 95.8 ± 9.7 | 0.172 |
| **Blood Test Results** | | | |
| Total cholesterol (mmol/l) | 5.2 ± 1.0 | 4.8 ± 0.9 | 0.001^+^ |
| Triglyceride (mmol/l | 1.5 ± 0.7 | 1.5 ± 0.8 | 0.642 |
| HDL (mmol/l) | 1.4 ± 0.3 | 1.2 ± 0.2 | < 0.001^+^ |
| LDL (mmol/l) | 3.2 ± 0.9 | 2.9 ± 0.8 | 0.018^+^ |
| Blood glucose level (fasting, mmol/l) | 5.3 ± 0.6 | 5.4 ± 0.5 | 0.150 |
| **SDB Variables** | | | |
| AHI (events/hr)^*^ | 8.0 (3.3 – 14.6) | 32.4 (22.1 – 45.3) | 0.000^+^ |
| RDI (events/hr)^*^ | 17.2 (11.8 – 25.0) | 44.5 (36.0 – 54.4) | 0.000^+^ |
| AI (events/hr)^*^ | 22.8 (16.7 – 28.9) | 42.5 (34.9 – 51.6) | 0.000^+^ |
| ODI >3% (events/hr)^*^ | 2.2 (0.7 – 5.7) | 13.7 (5.4 – 26.0) | 0.000^+^ |
| SaO2 <90% (% TST)^*^ | 0.1 (0.0 – 1.2) | 3.4 (1.0 – 9.7) | < 0.001^+^ |
| **Vessel Diameters** | | | |
| Evening CRAE (µm) | 144.04 ± 12.65 | 141.83 ± 13.64 | 0.203 |
| Evening CRVE (µm) | 213.92 ± 18.33 | 213.25 ± 20.24 | 0.793 |
| Evening AVR (a.u) | 0.68 ± 0.05 | 0.67 ± 0.05 | 0.190 |
| Morning CRAE (µm) | 144.95 ± 13.25 | 141.49 ± 14.58 | 0.061 |
| Morning CRVE (µm) | 215.69 ± 20.56 | 214.36 ± 20.63 | 0.629 |
| Morning AVR (a.u) | 0.67 ± 0.05 | 0.66 ± 0.05 | 0.122 |
| Delta CRAE (µm) | 0.70 ± 5.94 | -0.33 ± 5.32 | 0.182 |
| Delta CRVE (µm) | 2.04 ± 9.06 | 1.11 ± 8.87 | 0.444 |

Note: All variables tested via t-test; except * indicates Mann-Whitney test, ^+^ Indicates significant (p<0.05) difference.

SDB=Sleep Disordered Breathing, BMI = Body Mass Index; WHR = waist/hip ratio; BP = blood pressure; MAP = Mean Arterial Pressure; HDL = high-density lipoprotein; LDL = low-density lipoprotein; AHI = Apnea-Hypopnea Index; RDI = Respiratory Disturbance Index; AI = Arousal Index; ODI = Oxygen Desaturation Index; SaO2 = Oxygen Saturation; TST= total sleep time; CRAE = Central retinal arteriolar equivalent; CRVE = central retinal venular equivalent; AVR = arterio-venule-ratio; Delta = overnight change.

**B)**

| **Variable** | **Main Group**  **[n (%)]** | **RFM/CPAP Sub-Group**  **[n (%)]** | **p Value** |
| --- | --- | --- | --- |
| N *(Male %)* | 179 (48.6%) | 85 (63.5%) | 0.023 ^+^ |
| Ethnicity *(Caucasian)* | 170 (65.9%) | 81 (71.6%) | 0.365 |
| Hypertension history | 177 (38.4%) | 85 (54.1%) | 0.016 ^+^ |
| Hypercholesterolemia history | 174 (44.3%) | 84 (47.6%) | 0.611 |
| Smoking history | 174 (43.7%) | 85 (49.4%) | 0.384 |
| FRS risk level | 166 | 81 | 0.010 ^+^ |
| Low | (51.2%) | (37.0%) |  |
| Intermediate | (35.5%) | (34.6%) |  |
| High | (13.3%) | (28.4%) |  |
| AHI categories | 179 | 85 | < 0.001 ^+^ |
| Normal *(AHI <5 events/hr)* | (38.5%) | (3.5%) |  |
| Mild *(AHI 5-15 events/hr)* | (38.5%) | (14.1%) |  |
| Moderate *(AHI 15-30 events/hr)* | (15.6%) | (30.6%) |  |
| Severe *(AHI > 30 events/hr)* | (7.3%) | (51.8%) |  |
| OSA severity | 179 | 85 | < 0.001 ^+^ |
| Non-severe *(AHI < 30 events/hr)* | (92.7%) | (48.2%) |  |
| Severe *(AHI > 30 events/hr)* | (7.3%) | (51.8%) |  |

^+^ Indicates significant (p<0.05) differences using χ^2^ test.

FRS = Framingham risk score; OSA = Obstructive Sleep Apnea; AHI = Apnea-Hypopnea Index.
