# Supplementary material for: Sleep disordered breathing has minimal association with retinal microvascular diameters in a non-diabetic sleep clinic cohort
Source: PLoS One. 2023 Jan 10;18(1):e0279306. doi: 10.1371/journal.pone.0279306 (PMC9831323; doi:10.1371/journal.pone.0279306)
Supplement: S3 Table — Base Models showing non- SDB variable predictors for evening CRAE (A) and evening CRVE (B) for the Main Group. (DOCX) [file pone.0279306.s004.docx]

**Table S3: Multiple Linear Regression Models for Evening Retinal Vessel Diameters using Non -SDB Variables (Base Models) - Main Group (n=264)**

Base Models showing non- SDB variable predictors for evening CRAE (A) and evening CRVE (B) for the Main Group.

**A)**

| **Predictors** | **B** | **Std. Error** | **p** |
| --- | --- | --- | --- |
|  |  |  |  |
| **Retinal Arteriole Vessel Diameter (**µm**)** | | | |
| Constant | 217.304 | 17.645 | < 0.001 |
| Age (years) | -0.247 | 0.098 | 0.012 |
| Ethnicity (Caucasian) | -4.454 | 1.738 | 0.011 |
| Height (cm) | -0.245 | 0.086 | 0.005 |
| Total Cholesterol (mmol/l) | 1.318 | 0.825 | 0.112 |
| Smoking History | 3.085 | 1.578 | 0.052 |
| Systolic BP (mmHg) | -0.184 | 0.054 | < 0.001 |
| R^2^ = 0.178, p = < 0.001 | | | |

**B)**

| **Predictors** | **B** | **Std. Error** | **p** |
| --- | --- | --- | --- |
|  |  |  |  |
| **Retinal Venule Vessel Diameter (**µm**)** | | | |
| Constant | 265.610 | 25.841 | < 0.001 |
| Ethnicity (Caucasians) | -11.517 | 2.392 | < 0.001 |
| Age (years) | -0.235 | 0.133 | 0.078 |
| Height (cm) | -0.218 | 0.122 | 0.074 |
| Smoking history (ever smoked) | 4.616 | 2.165 | 0.034 |
| Hypertension history (present) | -6.517 | 2.285 | 0.005 |
| Systolic BP (mmHg) | -0.140 | 0.077 | 0.072 |
| Diastolic BP (mmHg) | 0.343 | 0.100 | 0.001 |
| R^2^ = 0.252, p = < 0.001 | | | |

SDB= Sleep Disordered Breathing, B = unstandardized beta coefficient; S.E. = standard error of B; R^2^ = Coefficient of determination; p = p value. BP = Blood pressure.
