# Supplementary material for: Sleep disordered breathing has minimal association with retinal microvascular diameters in a non-diabetic sleep clinic cohort
Source: PLoS One. 2023 Jan 10;18(1):e0279306. doi: 10.1371/journal.pone.0279306 (PMC9831323; doi:10.1371/journal.pone.0279306)
Supplement: S4 Table — Results of adding each SDB variable individually to the Base Model for evening CRAE (A) and evening CRVE (B) in the Main Group. (DOCX) [file pone.0279306.s005.docx]

**Table S4: Multiple Linear Regression Models for Evening Retinal Vessel Diameters using SDB Variables (SDB Models) - Main Group (n=264)**

Results of adding each SDB variable individually to the Base Model for evening CRAE (A) and evening CRVE (B) in the Main Group.

**A)**

| **Variables (Baseline model + …)** | **B** | **S.E** | **p value** | **R^2^** | **∆ R^2^** |
| --- | --- | --- | --- | --- | --- |
| **Retinal Arteriole Vessel Diameter (Evening CRAE,** µm**)** | | | | | |
| LnAHI + 1 (events/hr) | 0.562 | 0.752 | 0.455 | 0.180 | 0.002 |
| LnRDI (events/hr) | 0.019 | 0.979 | 0.984 | 0.178 | 0 |
| LnAI (events/hr) | 1.935 | 1.530 | 0.207 | 0.184 | 0.006 |
| LnODI >3% +1 (events/hr) | 0.345 | 0.745 | 0.644 | 0.179 | 0.001 |
| LnSaO_2_ < 90% + 1 (%TST) | 0.526 | 0.787 | 0.505 | 0.179 | 0.001 |
| AHI Category | - | - | 0.281 | 0.192 | 0.014 |
| AHI severity >30 events/hr | 3.373 | 1.950 | 0.085 | 0.188 | 0.010 |

**B)**

| **SDB Variables (Baseline model + …)** | **B** | **S.E** | **p value** | **R^2^** | **∆ R^2^** |
| --- | --- | --- | --- | --- | --- |
| **Retinal Venule Vessel Diameter (Evening CRVE,** µm**)** | | | | | |
| LnAHI + 1 (events/hr) | 0.174 | 1.017 | 0.864 | 0.252 | 0 |
| LnRDI (events/hr) | 0.906 | 1.340 | 0.500 | 0.253 | 0.001 |
| LnAI (events/hr) | 3.121 | 2.119 | 0.142 | 0.259 | 0.007 |
| LnODI >3% +1 (events/hr) | 0.239 | 1.022 | 0.815 | 0.252 | 0 |
| LnSaO_2_ < 90% +1 (%TST) | 0.378 | 1.105 | 0.733 | 0.252 | 0 |
| AHI Category | - | - | 0.449 | 0.260 | 0.008 |
| AHI severity >30 events/hr | 3.872 | 2.675 | 0.149 | 0.259 | 0.007 |

B = unstandardized beta coefficient; S.E. = standard error of B; ΔR^2^ is the change in R^2^ from the Base Model after addition of the SDB variable

AHI = Apnoea-Hypopnea Index; RDI = Respiratory Disturbance Index; AI = Arousal Index; ODI = Oxygen Desaturation Index; SaO2 = Oxygen Saturation.
