# Supplementary material for: Sleep disordered breathing has minimal association with retinal microvascular diameters in a non-diabetic sleep clinic cohort
Source: PLoS One. 2023 Jan 10;18(1):e0279306. doi: 10.1371/journal.pone.0279306 (PMC9831323; doi:10.1371/journal.pone.0279306)
Supplement: S5 Table — Results of adding LnAHI+1 and AHI Category into the Base Model for CRAE in the RFM/CPAP Sub-Group. (DOCX) [file pone.0279306.s006.docx]

**Table S5: Multiple Linear Regression Models for Evening CRAE using SDB Variables (SDB Models)- RFM/CPAP Sub-Group (n=85)**

Results of adding LnAHI+1 and AHI Category into the Base Model for CRAE in the RFM/CPAP Sub-Group.

| **Variables (Baseline model + …)** | **B** | **S.E** | **p value** | **R^2^** | **∆ R^2^** |
| --- | --- | --- | --- | --- | --- |
| **Retinal Arteriole Vessel Diameter (Evening CRAE,** µm**)** | | | | | |
| LnAHI + 1 (events/hr) | 4.603 | 2.063 | 0.029 | 0.253 | 0.053 |
| AHI Severity >30events/hr | 7.340 | 2.852 | 0.012 | 0.270 | 0.070 |

B = unstandardized beta coefficient; S.E. = standard error of B; ΔR^2^ is the change in R^2^ from the base model after addition of the SDB variable. AHI = Apnoea-Hypopnea Index
