# Supplementary material for: Sleep disordered breathing has minimal association with retinal microvascular diameters in a non-diabetic sleep clinic cohort
Source: PLoS One. 2023 Jan 10;18(1):e0279306. doi: 10.1371/journal.pone.0279306 (PMC9831323; doi:10.1371/journal.pone.0279306)
Supplement: S1 Study protocol — (PDF) [file pone.0279306.s011.pdf]

INTRODUCTION:

This proposal focuses on the mechanisms by which snoring and obstructive sleep apnoea syndrome (OSAS) may contribute to the development of cerebral small and large vessel disease leading to stroke. We will explore the general hypothesis that epidemiologically demonstrated links between OSAS and stroke are mediated, at least partially, through development of specific stroke promoting vascular pathologies. Retinal microvasculature morphology provides a direct window on the cerebral circulation for assessing the integrity of the microvasculature, while carotid artery morphology is an established indicator of stroke risk from macrovascular causes. We will utilise the accessibility of carotid and retinal blood vessels to test the hypothesis that:

Untreated heavy snoring and OSAS is associated with increased prevalence of cerebral microvascular disease (reflected via retinal vessel morphology and micro-emboli) and/or macrovascular disease (reflected via carotid intima-media thickness [IMT]).

The pathological mechanisms that lead to stroke in OSAS are unclear, but may include: 1) the development of cerebral microvascular disease (associated with lacunar stroke); 2) the development of carotid atherosclerosis (associated with thrombotic stroke); 3) cerebral embolic stroke from active carotid plaque disease (possibly due to snoring related carotid artery vibration); and 4) haemorrhagic stroke. Of these potential mechanisms, only the development of carotid atherosclerosis in snoring and OSAS has been investigated, with no studies exploring possible links to cerebral embolic disease and the development of cerebral microvascular disease.

Our specific hypotheses are:

1. Patients with untreated heavy snoring and OSAS will demonstrate an increased prevalence of retinal microvascular and carotid macrovascular abnormalities.

2. Microvascular and macrovascular abnormalities will increase over a night of sleep, with the overnight increase correlating with the severity of the snoring and OSAS.

3. Patients with untreated heavy snoring and OSAS, and with established carotid atherosclerotic plaque, will demonstrate an increased incidence of overnight retinal arteriolar emboli (compared with normal controls), consistent with active carotid plaque disease.

4. Early changes of cerebral microvascular disease (indicated by retinal abnormalities) and macrovascular disease (indicated by carotid atherosclerosis and IMT) present in patients with severe OSAS will regress following 12 months treatment with nasal CPAP therapy.

Project 1: ***In a cohort of 390 patients (aged >45years) with snoring and OSAS,*** we will: 1) use laboratory polysomnography (PSG) with monitoring of snoring sounds to categorise OSAS severity; 2) take retinal photographs before (pm) and after (am) sleep to quantify retinal microvascular lesions including incident retinal emboli; 3) quantify carotid artery IMT, atheromatous plaque and lumen diameter using Doppler ultrasound; 4) quantify cardiovascular risk factors before and after sleep. In untreated snoring and OSAS, Project 1 will establish: (i) the prevalence of retinal macrovascular and microvascular abnormalities (including retinal emboli); and (ii) the association between vascular abnormalities and disease severity.

Project 2: ***In a subgroup of 80 heavy snoring and OSAS patients, we will undertake a 12 month clinical trial of CPAP therapy***, following stabilisation of optimal standard therapy for cardiovascular risk factors. We will record diagnostic PSG with retinal photography (pm and am) and carotid artery ultrasound (pm) as a baseline. After institution of CPAP therapy, compliance will be monitored monthly, with repeat retinal photography (pm and am) and carotid artery ultrasound following 6 and 12 months of therapy. This study will establish if the addition of CPAP therapy will result in regression of cerebral microvascular disease (retinal abnormalities) and macrovascular disease (carotid atherosclerosis and IMT) present in patients with heavy snoring and OSAS.

To undertake this project we have an established and experienced CI/AI multidisciplinary research team incorporating expertise in clinical sleep medicine and physiology [CIA/CID/CIE],

clinical trials and stroke (CIB), retinal photography and cerebral microvascular disease (CIC and AIs Mitchell and Wong) and carotid ultrasonography (AI Larcos). Sleep studies will be undertaken in the sleep laboratory research facilities at Westmead Hospital (CIA/CID/CIE), and the retinal photography analysis will be performed in the Centre for Vision Research (CIC/AI Mitchell). Long term cohort follow-up will be co-ordinated by the George Institute, University of Sydney (CIB).

BACKGROUND:

Snoring and OSAS (sleep disordered breathing [SDB]) are a common consequence of increased upper airway resistance during sleep (3). Habitual snoring (every night or almost every night) without overt OSAS is highly prevalent in the community, occurring in approximately 40% of men and 20% of women (5, 65). The prevalence of OSAS in middle age is about 4% for men and 2% for women (65). Increasingly, it is recognized that snoring and OSAS pose real risks to health (21). Emerging data are highly suggestive of an independent role in the pathogenesis of stroke (28), hypertension (9), coronary (30) and carotid atherosclerosis (29). However, the mechanisms underlying this observed stroke risk are unknown with a wide variety of suggested possibilities. Understanding the mechanisms of this increased risk will be an important step to reduce the burden of this chronic disease arising in an ageing population with an increasing prevalence of obesity.

OSAS is now generally regarded as an independent risk factor for stroke (2, 37). Epidemiological data suggest a strong relationship between OSAS and acute cerebrovascular events. The prevalence of SDB in patients with acute stroke ranges 44-72%, and after the acute phase still remains higher than in the general population (64, 56). However, uncertainties on the role of OSAS in stroke pathogenesis relate to the observation that OSAS can both precede or follow stroke occurrence (19).

In a cross-sectional analysis of >6000 subjects from the Sleep Heart Health Study, the prevalence of stroke was 60% higher (odds ratio [OR] 1.58) among those subjects with OSAS with an apnoea-hypopnoea index (AHI) >11 events/hour (50). OSAS with an AHI of >20 /hour was associated with a 4-fold increased risk (OR 4.3) of suffering a first-ever stroke over a 4 year follow-up (2).

Pathogenic involvement of OSAS in cerebrovascular disease is suggested by the direct relationship found between the severity of nocturnal oxygen desaturation and carotid intima-media thickness (IMT) and/or the occurrence of atherosclerotic plaques in the carotid arteries of OSAS patients, independent of hypertension (4, 54). Recently, two studies in patients with untreated PSG verified severe OSAS (14, 41), at low risk from the classical cardiovascular risk factors, have demonstrated the presence of early carotid atherosclerosis (increase in IMT). Thus, patients with severe OSAS appear more likely to develop early changes of cerebral macrovascular disease.

We recently published a large cross-sectional study* (29) *in 110 heavy snorers (with only mild OSAS), with adequate power to assess associations between snoring and carotid vascular disease. The prevalence of carotid atherosclerotic plaque was 31%, with significant risk factors including age, male gender, hypertension, smoking history and heavy snoring. **The adjusted odds ratio for carotid atherosclerosis in the heavy snoring group (snoring greater than 50% sleep time) was substantially increased at 10.5 (2.1 – 51.8, 95% CI),** and was clearly the strongest association. Importantly, this study is the first to demonstrate that snoring with mild OSAS (without nocturnal hypoxia) is a strong independent risk factor for carotid atherosclerosis. In support of this, the prevalence of carotid plaque in this study population was 1.8 times greater than that reported for the general population (27), and even higher than that seen in OSAS populations (41).

The novelty and importance of this study by CIA and his team were recognised by the publication of two separate editorial commentaries in the journal *Sleep* (16, 36), both of which concluded that the relationship between snoring and carotid atherosclerosis is an exciting and important new area of research to elucidate factors contributing to cardiovascular disease risk profiles. They noted that the significance of the finding may elevate the importance of snoring from a social annoyance to a vascular risk factor. If the independent association between snoring and carotid atherosclerosis

observed in this study is indeed causal, the significantly increased risk of carotid atherosclerosis in heavy snorers, coupled with the high prevalence of snoring and mild OSAS in the community, has substantial public health implications for the prevention of stroke and cardiovascular disease.

Mechanisms that have been implicated in the increased risk of stroke in OSAS include blood pressure swings, reduction in cerebral blood flow, altered cerebral autoregulation, impaired endothelial function, accelerated atherogenesis, and prothrombotic and proinflammatory states (52). In broad terms, the occurrence of stroke may be associated with the following types of cerebral vascular disease: 1) cerebral microvascular disease (associated with lacunar stroke); 2) carotid atherosclerosis (associated with thrombotic stroke); 3) cerebral embolism from carotid plaque rupture (possibly due to carotid artery vibration) or cardiac thrombus dislodgement; and 4) cerebral haemorrhage. Of these potential mechanisms, only the prevalence of carotid atherosclerosis has been investigated, with no studies exploring the possible links of snoring and OSAS to carotid plaque embolism or the development of cerebral microvascular disease. One focus of this proposal is the role of both snoring and OSAS as causes of cerebral micro-emboli and microvascular disease, both important stroke mechanisms, that have not been previously investigated in these populations.

Retinal microvascular disease can now be readily quantified with digital retinal photography, and the qualitative and quantitative analysis of the retinal microvasculature has emerged as a powerful non-invasive tool for assessment of the cerebral microcirculation (53). Retinal photography has been revolutionised by advances in imaging and computer techniques that permit retinal microvascular signs to be non-invasively studied in great detail with computerised image processing techniques (62). High quality digital photographs of the retina can be obtained using a retinal camera. In recent years, reliable assessment methods have been developed and refined leading to a battery of qualitative and quantitative methods for evaluating retinal pathology (53).

Assessment of retinal vascular morphology is useful in two broad areas: providing clues to pathophysiological mechanisms and in risk stratification. The value in understanding pathophysiological mechanisms lies in the ability to directly view the microcirculation, and the similarities of the retinal to the cerebral circulation. There is a common embryological origin, a similar anatomical structure (non-anastomotic end-arteries) and both have a barrier to the blood (blood/brain; blood/retina barrier) (60, 62). In addition, many retinal signs have been found to have prognostic significance for stroke over and above recognised risk factors (32, 62).

Qualitative assessment of retinal vascular morphology includes: focal arteriolar narrowing, arteriovenous (AV) nicking, arteriolar wall opacity, retinopathy signs (retinal haemorrhages, microaneurysms and/or cotton wool spots), retinal emboli and retinal vein occlusion. Retinal emboli are intravascular lesions seen either along the course of retinal arterioles or at a bifurcation (42).

Quantitative assessment of retinal vascular morphology includes estimates of the internal calibre of retinal arterioles, venules and the arteriole-to-venule ratio (AVR). Recent large population-based studies have demonstrated that relative narrowing of retinal arteriolar calibre, or widening of retinal venular calibre, carry predictive significance for stroke (38, 39).

Data from a number of large population-based cohorts have provided new insights into the prognostic value of retinal microvascular signs (1, 17, 22, 25, 26, 43, 48, 58, 59, 63)**.** Thus, in total, there are now population-based data on the prognostic value of retinal signs in more than 30,000 people (62). Data from the ARIC study reported that individuals with a smaller AVR tend to have more white matter lesions (61), MRI cerebral infarcts (10) and an increased risk of incident clinical stroke (59). Prospective data from both the Rotterdam and Cardiovascular Health studies have consistently demonstrated that larger retinal venular calibre is associated with an increased clinical risk of stroke (25, 63). The Rotterdam study has further shown that wider venular calibre is associated with cerebral infarction (25), MRI-defined white matter lesions, and lacunar infarction (26). Thus, there is considerable evidence supporting an association of altered retinal vascular calibre with both clinical and sub-clinical stroke (53).

In summary, both arteriolar narrowing and venular widening are associated with clinical outcomes such as stroke. Narrowed arteriolar calibre and smaller AVR predict clinical stroke, and hence, retinal vascular calibre has great potential as a reflection of the cerebral vasculature to predict the risk of stroke (53). In this proposal, retinal photography will help to characterise the pathophysiology of the cerebral small vessel disease related to OSAS, identify whether retinal microvascular signs are prevalent in OSAS, and whether successful treatment with CPAP leads to reversal of retinal small vessel abnormalities with presumed reduction in stroke risk.

Retinal microvascular imaging has three potential roles in snoring and OSAS patients:

1) Exploring pathophysiological mechanisms of stroke. OSAS and stroke share several classical vascular risk factors but the exact mechanism(s) of snoring and OSAS associated stroke is not known. Potential mechanisms include accelerated small vessel disease, perhaps due to hypoxia or sympathetic blood pressure surges, but could also include physical factors such as snoring vibration induced/promoted atherosclerosis (20, 29, CIA18). Retinal microvascular signs provide a method of exploring these potential mechanisms. For example, increasing severity of OSAS may provoke characteristic small vessel changes that have been associated with lacunar stroke (33). Recent meta-analysis has demonstrated that retinal venular dilatation is associated with incident stroke, and retinal hypoxia is one postulated cause (39). A relationship between the severity of nocturnal hypoxia in OSAS and wider retinal vein diameter may provide additional evidence of the importance of hypoxia in the development of this sign. Recent work from our retinal group has demonstrated that retinal microvascular signs are associated with microvascular (lacunar) stroke, and, if found to be highly prevalent in an OSAS population, would provide important new evidence to explain that the excess stroke risk from this disorder is partly due to small vessel disease (33). Work from our sleep medicine group (29) has been focused on the ability of snoring vibrations to directly cause or accelerate carotid atheromatous disease (macrovascular disease related to stroke). Complicated atheroma is a source of microemboli, snoring vibrations have the potential to disrupt carotid plaque (20), and retinal photography has the potential to identify any resultant emboli.

2) Stroke risk stratification. As retinal microvascular signs may be a surrogate for the cumulative impact from life-long burden of vascular risk factors (32), the prevalence of signs may differ by severity of snoring and OSAS. This would allow additional vascular risk stratification in OSAS patients, and would help target intensive risk factor management to those at greatest vascular risk.

3) Surrogate outcomes. CPAP treatment for those with severe OSAS is largely offered for symptomatic relief, and until the results of current RCTs are available, it is unknown whether this treatment can prevent stroke, myocardial infarction or cardiovascular death. Repeated retinal imaging of those with heavy snoring and OSAS, treated with CPAP, may demonstrate reversal of retinal microvascular signs. These data will improve our understanding on how snoring and OSAS confer excess vascular risk, and strengthen the indications for CPAP therapy to reduce vascular risk.

***Only one published study has addressed the issue of retinal microvascular abnormalities in OSAS* (6).** The Sleep Heart Health Study examined 2,927 subjects, but only 1% of subjects had a clinical diagnosis of OSAS. Thus the study was underpowered to detect retinal abnormalities as a function of OSAS. Nevertheless, the overall prevalence of retinopathy was increased at higher AHI values, and an increase in AHI from 0 to 10 was associated with a decrease in the arteriole-to-venule ratio (AVR). The authors concluded that further investigation was warranted.

Preliminary analysis of data from the **Blue Mountains Eye Study** (BMES; CIC) demonstrated a large increase in venular calibre for a self reported history of OSAS (224.5μm) versus non affected non-snorers (219.7 μm; p=0.06), suggestive of early microvascular disease. Data from the **Wisconsin Sleep Cohort** (n=491) have demonstrated that a higher AHI was positively associated with wider retinal venular calibre (for AHI >15 events/hour, OR 2.08 [1.03-2.16]; p<0.05), independent of other cardiovascular risk factors (personal communication). This supports an association of SDB with microvascular mediated cerebrovascular disease.

Intima-Media Thickness (IMT) and Cerebral Macrovascular (large vessel) Disease

Carotid IMT is used as an intermediate phenotype for early atherosclerosis, is quantifiable on a linear scale, and is the primary macrovascular outcome variable in this study. Because it can be measured simply and non-invasively, it is well suited to larger clinical studies as an end point to stratify risk. Ultrasonic measurements correlate well with histology (46), and increased IMT is associated with vascular risk factors (7, 8, 49) and the presence of more advanced atherosclerosis (44). ***A systematic review and meta-analysis demonstrated that carotid IMT is a strong predictor of future vascular events (34),*** with an adjusted relative risk of stroke of 1.18 (95% CI, 1.16 to 1.21) per 0.10 mm common carotid artery IMT increase. Our quantification of IMT will follow standardised measurement methodology (55). Ultrasound will be performed by two experienced sonographers under the direction of AI Larcos, thus minimising between-technician variation.

The potential for microvascular and macrovascular disease regression with removal of a stimulus has been confirmed in several studies. For example, antihypertensive treatment in 25 patients with untreated hypertension over one year was associated with a reduction in retinal arteriolar narrowing, a widening of arteriolar branch angle and an increase in arteriolar density (24). In another study of 51 hypertensive patients, 6 months of antihypertensive treatment was associated with a reduction in mean retinal arteriole diameter of 0.5 μm and a corresponding increase in AVR (47). Thus regression of retinal microvascular disease with effective treatment is a plausible outcome. In addition, there are now several clinical studies that have demonstrated reductions in carotid IMT (indicative of regression of early atherosclerosis) over a six to twelve month period with statin lipid profile altering medications (18, 35, 40). These studies have consistently demonstrated significant regression or slowing of progression of carotid IMT with therapy, and benefits were evident after as little as six to twelve months (40).

If OSAS constitutes an independent stimulus to the development of cerebral microvascular and macrovascular disease, it is plausible that removal of this stimulus with CPAP therapy may also be associated with regression of the early changes of vascular disease within the six to twelve month time frame. In support of this, a recent study by Drager et al (15) demonstrated that effective treatment of a group of 12 severe, hypoxic OSAS patients with CPAP for 4 months resulted in a significant decrease in carotid IMT of 0.063mm, compared with a small increase of 0.008mm in 12 control OSAS subjects. Overall, this represented a 9% reduction in IMT over only a four month treatment period. This study provides strong support that CPAP treatment is able to significantly reduce the severity of cerebral macrovascular disease within a reasonable timeframe. We propose to test this proposition in order to establish the benefits of CPAP treatment in terms of both cerebral microvascular and macrovascular risk reduction in patients with OSAS.

Preliminary Data: We have completed a pilot study in 28 subjects referred for investigation of SDB, with retinal photography performed before and after one night of PSG. Subject characteristics: age 55.1±11.9 years (mean±SD); BMI 34.4±7.3 kg/m^2; RDI 31.7±24.9 events/hour (range 0-102); snoring 49.4±26.2 % sleep epochs. The prevalence of retinal emboli was 10.7% (present in 3/28 subjects both pre and post sleep; all 3 with heavy snoring or severe OSAS). This represents an 8-fold increase in prevalence of asymptomatic retinal emboli compared with population data from the local BMES (prevalence 1.4%; 95% CI 1.0 to 1.8%) (42). Overnight, mean retinal arteriolar diameter decreased from 153.9±13.5 to 151.7±14.2 μm ($p<0.02$); mean venular diameter increased from 222.9±23.2 to 227.3±22.5 μm ($p<0.016$); and mean AVR fell from 0.69±0.06 to 0.67±0.06 ($p=0.001$; Fig 1). Correlation analysis (Spearman's) demonstrates significant associations between: (i) increasing snoring time and a decrease in retinal arteriolar diameter ($r = 0.42$, $p<0.03$; Fig 2); and (ii) overnight oxygen desaturation and decreasing AVR ($r=0.38$, $p<0.05$). In the subjects where RDI was <15 events/hour (control), there was no change in overnight retinal arteriolar or venular diameters ($p>0.5$). Thus, preliminary data demonstrate that there is: 1) no overnight change (sleep or circadian influence) in retinal microvasculature in control/mild OSAS subjects; 2) a clear overnight increase in venular diameter, and decrease in

arteriolar diameter and AVR in OSAS subjects; 3) a decrease in arteriolar diameter which is associated with increasing snoring time; and 4) an increased prevalence of retinal emboli in OSAS. These are the first data to demonstrate overnight change in the retinal microvasculature.

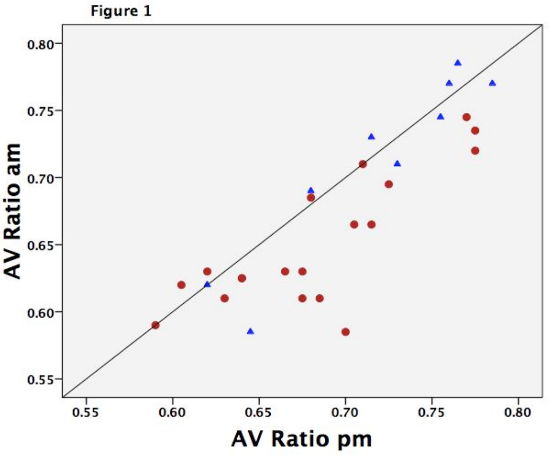

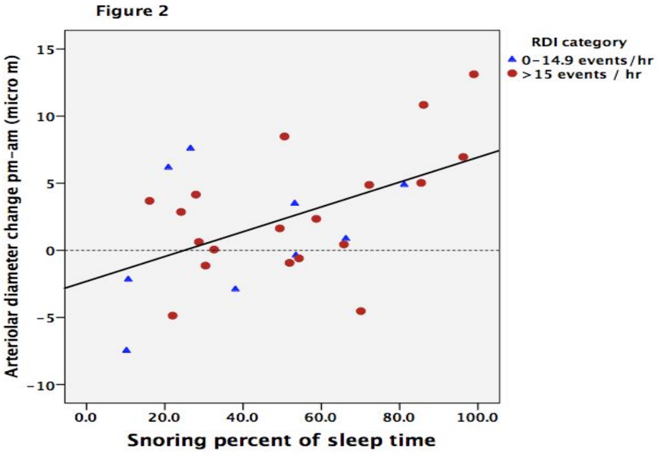

Figure 1: Identity plot of AV Ratio am versus pm. Note that controls (triangles) fall along the identity line; cases (circles) are shifted to the right, consistent with overnight decrease in AVR. Figure 2: Change in arteriolar diameter (pm-am) plotted against snoring. Note the correlation (solid line; $r=0.38$; $p<0.05$) between increasing snoring and decrease in overnight arteriolar diameter.

RESEARCH PLAN:

PROJECT 1: The Influence of Untreated Snoring and OSAS on Cerebral Vascular Disease.

Hypotheses: *1) Patients with untreated heavy snoring and OSAS will demonstrate an increased prevalence of retinal microvascular and carotid macrovascular abnormalities.*
2) Microvascular and macrovascular abnormalities will increase over a night of sleep, with the overnight increase correlating with the severity of the snoring and OSAS.
3) Patients with untreated heavy snoring and OSAS, and with established carotid atherosclerotic plaque, will demonstrate an increased incidence of overnight retinal arteriolar emboli (compared with normal controls), consistent with active carotid plaque disease.
We propose to address the above hypotheses by comparing pre and post sleep data in patients with untreated heavy snoring and OSAS and comparing any differences to those with mild or no snoring and OSAS, to directly address the following specific research questions:
1) Do patients with heavy snoring and OSAS demonstrate an increased baseline prevalence of microvascular disease (retinal vessel changes) and/or macrovascular disease (carotid artery IMT)?
2) Do patients with OSAS demonstrate a decrease in arteriolar diameter or an increase in retinal venular diameter following overnight sleep as a function of the severity of their OSAS or snoring?
3) Do patients with heavy snoring and OSAS, and with carotid atherosclerotic plaque, demonstrate a higher frequency of prevalent and overnight incident retinal arteriolar emboli?

Design: Nested case-control cohort study in snoring and OSAS referrals, comparing severe cases with control/mild cases (see below) at baseline and for overnight changes in vascular abnormalities.

Subjects: We will recruit 390 patients (130 patients/year) aged >45 years who are referred to the Westmead Hospital Sleep Laboratory for a routine diagnostic PSG for assessment of possible SDB. Exclusion criteria include past history of stroke, diabetes, atrial fibrillation, congestive cardiac failure, carotid artery surgery, and eye pathologies that preclude the measurement of retinal vessel calibre (e.g. age-related macular degeneration, glaucoma). Our Laboratory performs over 500 diagnostic PSG studies/year in this age group, with results demonstrating a Respiratory Disturbance Index (RDI) of <5 events/hr in 7% (normal controls), 5-15 events/hr in 24% (mild OSAS), 15-30 events/hr in 27% (moderate OSAS) and >30 events/hr in 42% (severe OSAS). Review of our

database indicates that 82% of target patients meet the inclusion criteria. We will stratify patient recruitment across the severity range. Allowing for a 50% participation refusal, there will be no problems recruiting target numbers.

Baseline/Anthropometry Data: We will record age, gender, body mass index, waist-hip ratio, and neck circumference. Questionnaire data will include cardiovascular risk (Framingham Questionnaire), Epworth Sleepiness Scale (ESS), snoring history, and smoking history. Detailed history of hypertension will include length of known diagnosis and current treatment. A fasting blood sample will be collected for lipid profile and glucose level.

Procedures: *PSG:* Sleep will be monitored using standard PSG proceedures for our laboratory (29). In addition, we will record snoring sounds using a room sound level meter and quantify snore-related tissue vibration energy using an accelerometer attached to the skin surface of the neck.
Carotid Artery Ultrasound: Doppler ultrasound examination of both carotid arteries will be used to measure IMT (57) of the common carotid, plaque type and extent, lumen diameter, and peak systolic velocity.
Retinal Photography: Each eye will be photographed following a standardized protocol using a high-resolution digital camera (Canon non-mydriatic). Two colour retinal photographs will be taken from each eye, one centred on the optic disc (Diabetic Retinopathy Study [DRS] standard field 1) and another on the macula (DRS standard field 2) **(**13**)**. Digital images will be graded for retinal microvascular lesions using standard protocols under direction of CIC. Qualitative signs will be recorded (see Background p3). The intra-grader reliability for detecting focal arteriolar narrowing and AV nicking is (kappa statistic) 0.80 and 0.87, respectively (57).
A computer-assisted grading method with high reproducibility will be used to measure the retinal vessel diameters and arteriole-to-venule ratio (AVR). Average retinal arteriolar or venular width (diameter) is calculated using the Parr-Hubbard formula (23, 45) and presented as the central retinal arteriolar equivalent (CRAE) or central retinal venular equivalent (CRVE). AVR can then be calculated from CRAE and CRVE. Intra- and inter-grader reliability of this method is high (51). Retinal emboli are intravascular lesions seen along the course of retinal arterioles, and may be present at a bifurcation. Detailed methods of grading retinal abnormalities have been described (57).

Protocol: Subjects will undergo an overnight PSG study. Carotid ultrasound, retinal photography and supine blood pressure will be performed at ~2000hrs and repeated at ~0600hrs. A major outcome focuses on overnight change in measurements, where subjects act as their own controls.

Statistical Analysis and Sample Size: Univariate relationships between measures of OSAS or snoring severity (AHI, Arousal Index, Oxygen Desaturation Index, snore index) **[**independent variables], and overnight change in CRAE, CRVE, AVR and IMT [dependent variables], will be examined using linear regression analysis for continuous data and X^2 analysis for categorical data. Stepwise multiple linear regression or logistic regression will be used to examine associations of OSAS and snoring (continuous data) with dependent variables, including any interactions with anthropometric and vascular risk factor data. From our preliminary data, a sample size of 384 for the cohort study will have 80% power to detect a difference in the CRVE means of 8.0 μm (and difference in CRAE means of 13.6 μm), assuming that the common standard deviation is 24.1 μm for CRVE and 13.6 μm for CRAE, using a two group t-test with two sided significance of p=0.017. For the overnight changes, a sample size of 146 will have 80% power to detect a correlation co-efficient of 0.25 between AVR and snoring using linear regression, assuming normal distribution of AVR and a two sided significance level of p=0.025. Power estimates are conservative based on the dependent variables with the smallest detected signals from our preliminary retinal data.

Outcomes: This study will establish the relationship between snoring / OSAS and the presence of cerebral microvascular and macrovascular disease in a cross section of subjects. Overnight changes in the retinal vessel calibre will demonstrate for the first time a mechanism by which SDB acutely and adversely affects the microvasculature. In addition, if the cross-sectional analysis of subjects

demonstrates the same changes in vessels as a function of SDB severity, then this would support these changes becoming chronic over time. Finally, we will examine for the presence of retinal emboli before and after sleep, which will provide the first evidence for snoring and OSAS being associated with active plaque disease, with potential for plaque disruption and embolic stroke.

PROJECT 2: Regression of Cerebral Vascular Disease in Severe OSAS using CPAP Therapy

Hypothesis: *Early changes of cerebral microvascular disease (indicated by retinal abnormalities) and macrovascular disease (indicated by carotid atherosclerosis and IMT) present in patients with severe OSAS will regress following 12 months treatment with nasal CPAP therapy.*

Design: This will be a one-year prospective, longitudinal, observational study of OSAS patients (acting as their own controls). Following discussion with our Ethics Committee, it would not be acceptable to have a randomised, controlled design with a sham treatment arm over a one year period for patients with severe, symptomatic OSAS.

Subjects: We will recruit 80 patients from Project 1 (40 in year 1 and 40 in year 2), who have been diagnosed with severe, symptomatic OSAS (RDI >30 events/hr, ESS >9). Exclusion criteria are as for Project 1, plus current smoking or an inability to tolerate CPAP therapy. Successive patients from Project 1 who meet the inclusion criteria will be approached to participate. Over the first two years, our laboratory will diagnose over 400 patients who meet the inclusion criteria.

Baseline/Anthropometry Data: Same as for Project 1.
Procedures: PSG, Carotid Artery Ultrasound, Retinal Photography as for Project 1.

Protocol: Recruited subjects (n=40/year for first 2 years) will complete a run-in period, with review of clinical history, blood lipids, and blood pressure to ensure that subjects have been stabilised on optimal standard therapy for cardiovascular risk factors (normal values with no clinically significant changes). Optimal standard therapy will include continued non-smoking, maintenance of weight, medication (as required) to keep daytime blood pressure and blood lipid profile normal. When clinically stable, diagnostic PSG with repeat carotid Doppler ultrasound (pm) and retinal photography (pm and am) will be performed as a baseline. Subjects will then have a laboratory CPAP titration study, to eliminate snoring and obstructive events as per laboratory protocol.
The study treatment period will run for 12 months, with monthly monitoring of CPAP compliance (machine hours at pressure recorded), regular study co-ordinator follow up, and continued monitoring and treatment of cardiovascular risk factors. Subjects will be stratified during analysis according to medication use for cardiovascular risk factors and CPAP compliance.
Overnight CPAP review studies using the patient's CPAP treatment pressure will be undertaken at 6 months and one year. Repeat carotid Doppler ultrasound (pm), retinal photography (pm and am), blood pressure (pm and am), fasting blood lipids and sugar, and weight will be measured.
This duration of study is adequate to detect clinically relevant outcomes. An increase in IMT of >0.03 mm/year is detectable and clinically relevant, as it is associated with a doubling in incidence of coronary events (44). We anticipate a reduction of at least this amount (15). For retinal photography, a decrease in 3.5 μm/year of arteriolar calibre is detectable, measurable in a one year time frame, and associated with a 10 mmHg increase in mean arterial blood pressure (31), making it clinically relevant. Both arteriolar narrowing and venular widening are predictive of stroke (59).

We have chosen to compare the same subjects before vs after the intervention but not to have an untreated control arm in this study due to efficiency of paired comparison and the well recognised ethical problems with not treating severe OSAS. This will enable a valid paired comparison with the differences between the before and after treatment measures being related to the treatment intervention over 1 year. The primary outcome variables (CRAE, CRVE, AVR and IMT) are objective measures of structural changes which are unlikely to spontaneously improve without intervention, or be affected by a placebo type response.

We acknowledge that compliance with CPAP treatment will be a critical factor to the success of this trial. Patients will be offered as much support as required in the initial phases of the trial by the study co-ordinator, with four weekly review throughout the study. Patients will be able to contact the study co-ordinator at any time to resolve problems. CPAP compliance will be monitored objectively by nights and hours of use from machine downloads at study visits, together with compliance with routine medication(s). We will target a minimum compliance of 4 hours per night.

Statistical Analysis and Sample Size: Power analysis is based on data relating to the observed reduction in IMT during CPAP therapy (15). Power calculations show that with a **sample size of 64** we will have 80% power to detect a difference in IMT of 0.025 mm over 12 months, assuming that the common standard deviation is 0.050 mm and using a two group t-test with a 0.05 two-sided significance level. Given the demonstrated reduction in IMT of 9% (0.063 mm) in 24 patients over only four months of CPAP treatment (15), we believe that our power calculation is quite conservative, and that our sample size is robust. Similarly, following 12 months of treatment for hypertension, there have been demonstrated improvements in retinal vessel diameter of 12.5% (24), consistent with the changes in IMT. We will study 80 subjects to allow for a 20% drop-out rate.

Significance: A reduction in atherosclerosis (decrease in IMT) and retinal microvascular changes (increase in CRAE, decrease in CRVE) will be related to CPAP therapy, as other cardiovascular risk factors will be stable over the study period, and any other functional changes will be adjusted for during analysis. ***This study will determine if CPAP treatment in patients with severe OSAS results in regression of both cerebral microvascular and macrovascular disease***, implicating OSAS as a pathogenic factor in cerebral vascular disease, and implying a possible role for treatment of OSAS in risk reduction from cerebral vascular disease.

FUTURE DIRECTIONS: Long-term risk of stroke in Heavy Snoring and OSAS Population

Hypothesis: *Long-term lifetime follow-up of a cohort of well-described patients with heavy snoring and OSAS will confirm the excess risk of stroke, and the predominant stroke subtypes.*

We intend to follow patients recruited in this project over their lifetime. Consent for lifetime follow-up for mortality of the subjects recruited in these projects will allow us to determine the risk of stroke. We will obtain date and cause of death via data linkage with the Australian National Death Index, and also obtain subsequent stroke events from the Centre for Health Record Linkage (CHeReL), ***which will not require additional NHMRC funding.*** Our current national follow-up for participants in RCTs (12) allows a cost-effective system of collecting events. This type of follow-up is inexpensive and can be sustained over time by CIA/CIB. There would be a pre-specified plan to continue follow-up at regular future intervals.

OUTCOMES AND SIGNIFICANCE:

These two projects investigate the macrovascular and microvascular mechanisms of the associations between both heavy snoring and OSAS with an increased risk of stroke. The use of retinal photography (for microvascular disease) and carotid ultrasound IMT (for macrovascular disease) are exciting new investigations which may provide practical clinical tools for stratification of overall cardiovascular risk in snoring and OSAS subjects. In addition, the second study will provide evidence supporting the benefit of CPAP treatment in reduction of cerebral microvascular and macrovascular disease, with consequent stroke risk reduction. Given the high prevalence of snoring and OSAS in the increasingly obese middle to older aged population, findings from these studies will demonstrate a new target for interventions to lower cardiovascular disease risk profiles in the population, and to further reduce the disease burden and premature deaths from stroke and other cardiovascular events.

Professor Paul Mitchell is an ophthalmologist and Director of the Centre for Vision Research, Westmead Millennium Institute, University of Sydney and leads a research program focused on the use of retinal imaging to predict risk of cardiovascular diseases, such as stroke and coronary heart disease. He will work closely with all the CIs in study design, particularly in the area of retinal vascular imaging, and in the analysis of the data, and interpretation of results. As Director of the Department of Ophthalmology, he will take responsibility for diagnosis of any pathological findings on the retinal photographs, that need referral.

Professor Tien Wong is an ophthalmologist and currently Professor at the Centre for Eye Research Australia, University of Melbourne and leads a research programme focused on the use of retinal imaging to predict risk of cardiovascular diseases, such as stroke and coronary heart disease. He will work closely with all the CIs in design of the study, particularly in the area of retinal vascular imaging, and in the analysis of the data, interpretation of results, and as a co-author in manuscripts.

Assoc Professor Peter Middleton is a senior staff specialist in the Department of Respiratory and Sleep Medicine at Westmead Hospital and a senior researcher in the Ludwig Engel Centre for Respiratory Research. For this project, Prof Middleton will assist with the recruitment of subjects, the analysis of polysomnography studies, data analysis and manuscript writing. A particular responsibility will be assisting with supervision of patients on the CPAP clinical trial.

Assoc Professor George Larcos is the Director of Nuclear Medicine and Ultrasound at Westmead Hospital. For this project Dr Larcos will supervise the collection, analysis and interpretation of all ultrasound data. A principal responsibility will be ensuring that ultrasound data collection is of the highest quality. He will assist in data analysis and manuscript writing.

REFERENCES

1. ARIC investigators. The Atherosclerosis Risk in Communities (ARIC) Study: design and objectives. Am J Epidemiol. 129:687-702, 1989.
2. Artz M *et al.* Association of SDB and occurrence of stroke. *AJRCCM.* 172: 1447-1451, 2005.
3. Ayappa I *et al*. The upper airway in sleep. *Sleep Med. Rev*. 7(1):9-33, 2003.
4. Baguet JP *et al.* The severity of oxygen desaturation is predictive of carotid wall thickening and plaque occurrence. *Chest.* 128: 3407-3412, 2005.
5. Bearpark H *et al.* Snoring and sleep apnoea. *AJRCCM.* 151(5): 1459-1465, 1995.
6. Boland LL *et al.* Sleep disordered breathing is not associated with the presence of retinal microvascular abnormalities: The Sleep Health Heart Study. *Sleep.* 27: 467-73, 2004.
7. Bots ML *et al*. Common carotid IMT and risk of stroke and myocardial infarction: the Rotterdam Study. *Circulation*. 96:1432-1437, 1997.
8. Chambless LE *et al*. Carotid wall thickness is predictive of incident clinical stroke: The Atherosclerosis Risks in Communities (ARIC) Study. *Am. J. Epidemiol.* 151:478-487, 2000.
9. Cho N *et al*. Relation of habitual snoring with components of metabolic syndrome in Korean adults. *Diabetes Res. Clin. Pract.* 71(3): 256-263, 2006.
10. Cooper LS *et al.* Retinal microvascular abnormalities and MRI-defined subclinical cerebral infarction: the Atherosclerosis Risk in Communities Study. *Stroke.* 37: 82-86, 2006.
11. Cugati S, **Wang JJ**, Rochtchina E, **Mitchell P**. 10-Year Incidence of retinal emboli in an Older Population. *Stroke.* 37: 908-910, 2006.
12. Dennis M, **Lindley R,** *et al.* Effectiveness of thigh-length graduated compression stockings to reduce the risk of deep vein thrombosis after stroke (CLOTS trial 1). *Lancet.* 373: 1958-1965, 2009.
13. Diabetic Retinopathy Study Research Group. DRS Report Number7: A modification of the Airlie House classification of diabetic retinopathy. *Investigative Ophthalmology & Visual Science*. 21: 210, 1981.
14. Drager LF *et al*. Early signs of atherosclerosis in OSA. *AJRCCM.* 172(5): 613-618, 2005.
15. Drager LF *et al.* Effects of CPAP on early signs of atherosclerosis in OSA. *AJRCCM* 176: 706-712, 2007.
16. Drager LF *et al.* Heavy snoring and carotid atherosclerosis: is there more than an association? *Sleep* 31(10): 1335, 2008.
17. Fried LP, Borhani NO, Enright P, *et al.* The Cardiovascular Health Study: design and rationale. *Ann Epidemiol*. 1: 263-76, 1991.
18. Furberg CD, et al. Effect of lovastatin on early carotid atherosclerosis and cardiovascular events. *Circulation.* 90(4):1679-1687, 1994.
19. Gibson GJ. Sleep Disordered Breathing and the outcome of stroke. *Thorax.* 59: 361-363, 2004.
20. Hedner J *et al.* Speculations on the interaction between vascular disease and OSA. Sleep and Breathing. Edited: Saunders NA and Sullivan C, New York: Dekker 1994: 823-846.
21. Hoffstein V. Is snoring dangerous to your health? *Sleep.* 19 (6):506-516. 1996.
22. Hofman A, Grobbee DE, de Jong PT, van den Ouweland FA. Determinants of disease and disability in the elderly: the Rotterdam Elderly Study. *Eur J Epidemiol.* 7: 403-22, 1991.
23. Hubbard LD, *et al*. Methods for evaluation of retinal microvascular abnormalities associated with hypertension/sclerosis in the Atherosclerosis Risk in Communities Study. *Ophthalmology*. 106: 2269-2280, 1999.
24. Hughes AD *et al.* Effect of antihypertensive treatment on retinal microvascular changes in hypertension. *Journal of Hypertension*. 26: 1703-07, 2008.
25. Ikram MK *et al.* Retinal Vessel diameters and risk of stroke: the Rotterdam Study. *Neurology.* 66: 1339-43, 2006.
26. Ikram MK *et al*. Retinal vessel diameters and cerebral small vessel disease: the Rotterdam Scan Study. *Brain.* 129: 182-8, 2006.

27. Irace C *et al*. Wall shear stress is associated with intima-media thickness and carotid atherosclerosis in subjects at low coronary heart disease risk. *Stroke.* 35(2): 464-468, 2004.
28. Koskenvuo M *et al*. Snoring as a risk factor for IHD and stroke in men. *Br. Med. J. (Clin. Res. Ed.).*294: 643, 1987.
29. Lee SA, Amis TC, Byth K, Larcos G, Kairaitis K, Robinson TD, **Wheatley JR**: Heavy snoring as a cause of carotid artery atherosclerosis. *Sleep.* 31:1207-1213, 2008.
30. Leineweber C *et al.* Snoring and progression of coronary artery disease. *Sleep.* 27(7):1344-1349, 2004.
31. Leung H, **Wang JJ, Mitchell P** *et al*. Relationships between age, blood pressure, and retinal vessel diameters in an older population. *Invest Opthalmol Vis Sci.* 44: 2900-04, 2003.
32. Liew G, **Wang JJ, Mitchell P,** Wong TY. Retinal vascular imaging: A new tool in microvascular disease research. *Circulation Cardiovascular Imaging.* 1: 156-161, 2008.
33. **Lindley RI, Wang JJ, Mitchell P** *et al.* Retinal microvasculature in acute lacunar stroke: a cross-sectional study. *Lancet Neurology*. 8: 628-34, 2009.
34. Lorenz MW *et al*. Carotid IMT indicates a higher vascular across a wide age range: prospective data from the Carotid Atherosclerosis Progression Study. *Stroke*. 37: 87-92, 2006.
35. MacMahon S, et al. Effects of lowering average of below-average cholesterol levels on the progression of carotid atherosclerosis. *Circulation.* 97(18):1784-1790, 1998.
36. Malhotra *et al.* Not so good vibrations. *Sleep*. 31(9):1207-1213, 2008.
37. Marin JM *et al*. Long-term cardiovascular outcomes in men with OSAH with or without treatment with continuous positive airway pressure. *Lancet.* 365(9464): 1046-1053, 2005.
38. McGeechan K, **Wang JJ, Mitchell P**, *et al.* Retinal vessel caliber and prediction of coronary heart disease: A systematic review and meta-analysis. *Annals of Internal Medicine.* 151:404-413, 2009.
39. McGeechan K, **Wang JJ, Mitchell P**, *et al*. Prediction of incident stroke events based on retinal vessel caliber: A systematic review and individual participant meta-analysis. *American Journal of Epidemiology.* 170:1323-1332, 2009.
40. Mercuri M, et al. Pravastatin reduces carotid IMT progression in an asymptomatic hypercholesterolemic Mediterranean population. *Am. J. Med.* 101(6):627-634, 1996.
41. Minoguchi K *et al.* Increased carotid IMT in OSA. *AJRCCM.* 172: 625-30, 2005.
42. **Mitchell P, Wang JJ**, Li W, Leeder SR, Smith W. Prevalence of asymptomatic retinal emboli. *Stroke.* 28: 63-66, 1997.
43. **Mitchell P, Wang JJ,** Wong TY, Smith W, Klein R, Leeder SR. Retinal microvascular signs and risk of stroke and stroke mortality. *Neurology*. 65: 1005-9, 2005.
44. O'Leary DH *et al*. Carotid-artery IMT as a risk factor for MI and stroke in older adults. Cardiovascular Health Study Collaborative Research Group. *N. Eng. J. Med.* 340:14-22, 1999.
45. Parr JC, Spears GF. General caliber of the retinal arteries expressed as the equivalent width of the central retinal artery. *Am J Ophthalmol*. 77: 472-477, 1974.
46. Pignoli P *et al.* Intimal plus medial thickness of the arterial wall: a direct measurement with ultrasound imaging. *Circulation*. 74:1399-1406, 1986.
47. Pose-Reino A *et al.* Regression of Alterations in Retinal Microcirculation Following Treatment for Arterial Hypertension. *J Clin Hypertens*. 8: 590-95, 2006.
48. Rosamond WD, *et al.* Stroke incidence and survival among middle-aged adults: 9-year follow-up of the Atherosclerosis Risk in Communities (ARIC) cohort. *Stroke*. 30: 736-43, 1999.
49. Rosvall M *et al.* Incidence of stroke is related to carotid IMT even in the absence of plaque. *Atherosclerosis*. 179: 235-331, 2005.
50. Shahar E et al. Sleep-disordered breathing and cardiovascular disease: cross-sectional results of the Sleep Heart Health Study. Am J Resp Crit Care Med. 163: 19-25, 2001.
51. Sherry LM, **Wang JJ**, *et al.* Reliability of computer-assisted retinal vessel measurement in a population. *Clinical & Experimental Ophthalmology*. 30: 179-82, 2002.

52. Somers VK *et al.* AHA/ACC Sleep Apnea and Cardiovascular Disease. *Journal of the American College of Cardiology*. 52: 686-717, 2008.
53. Sun C, **Wang JJ** *et al.* Retinal Vascular Caliber: Systematic, Environmental, and Genetic Associations. *Survey of Ophthalmology*. 54: 74-95, 2009.
54. Suzuki T *et al.* Obstructive sleep apnea and carotid artery intima-media thickness. *Sleep.* 27: 129-133, 2004.
55. Touboul PJ *et al.* Mannheim carotid IMT consensus. *Cerebrovasc. Dis.* 23:75-80, 2007.
56. Turkington PM *et al.* Sleep Disordered Breathing following stroke. *Monaldi Arch Chest Dis.* 61: 167-161, 2004.
57. **Wang JJ, Mitchell P** *et al.* Hypertensive retinal vessel wall signs in a general older population: the Blue Mountains Eye Study. *Hypertension.* 42: 534-41, 2003.
58. **Wang JJ,** Liew G, Klein R, *et al.* Retinal vessel diameter and cardiovascular mortality: pooled data analysis from two older populations. *Eur Heart J*. 28: 1984-92, 2007.
59. Wong TY *et al.* Retinal microvascular abnormalities and incident stroke: the Atherosclerosis Risk in Communities Study. *Lancet.* 358: 1334-40, 2001.
60. Wong TY *et al.* Retinal microvascular abnormalities and their relationship with hypertension, cardiovascular disease, and mortality. *Surv Ophthalmology.* 46: 59-80, 2001.
61. Wong TY *et al.* Cerebral white matter lesions, retinopathy, and incident clinical stroke. *JAMA.* 288: 67-74, 2002.
62. Wong TY. Is retinal photography useful in the measurement of stroke risk? *Lancet Neurol*. 3: 179-183, 2004.
63. Wong TY *et al.* Quantitative retinal venular caliber and risk of cardiovascular disease in older persons: the cardiovascular health study. *Arch Intern Med.* 166: 2388-94, 2006.
64. Yaggi H *et al.* OSA and stroke. *Lancet Neurology.* 3(6): 333-342, 2004.
65. Young T *et al.* Occurrence of SDB in middle-aged adults. *NEJM.* 328(17):1230-1235, 1993.

ID: 570789. ***Title:*** The Role of Snoring Vibrations in the Pathogenesis of Early Carotid Artery Atherosclerosis. ***Chief Investigators:*** JR Wheatley, TC Amis, P Witting

This project addresses the hypothesis that snoring induces a series of vibratory pressure waves in the tissue surrounding the pharyngeal airway, that are then transmitted to the carotid artery walls, leading to the development of carotid atherosclerosis through pathogenic effects on the carotid artery endothelium such as endothelial dysfunction. At the end of the second year of funding, all animal studies have been completed, and human studies are in the final stages of data collection. Six abstracts of work completed have been presented, and two manuscripts completed with one publication in 2010 and the second accepted for 2011. Studies undertaken during 2009-2010:

Project 1a: Natural snoring and carotid artery blood flow in heavy snorers
Preliminary studies have been completed in three snorers demonstrating that the technique is feasible, and relevant data can be gathered.
Project 1b: Simulated snoring and human carotid artery blood flow dynamics
This project uses carotid artery ultrasound to measure blood flow and an oesophageal balloon to monitor pleural pressure during simulated snoring and inspiratory resistive loading at different target levels. Studies have now been completed in six healthy subjects. Loud snores reduce carotid blood flow and wall shear stress. Further normal controls and heavy snorers will be recruited.
Project 1c: Snoring vibrations and local carotid artery blood flow responses:
In 10 anaesthetised rabbits, induced snoring and directly applied snoring-like vibrations were studied to assess their impact on carotid artery blood flow and vessel wall shear stress. Induced snoring reduced blood flow and wall shear stress. The direct application of vibrations did not result in any change in carotid artery bulk flow or diameter during vibration.
Project 2a: Snoring-like vibration of the carotid artery wall causes arterial endothelial injury
Studies have been completed in 10 anaesthetised rabbits. See Publication number 1.
Project 2b: Snoring-like vibration of the carotid artery wall causes endothelial dysfunction
Studies have been completed in 27 anaesthetised rabbits. See publication number 1. These data confirm the hypothesis that snoring vibrations can lead to endothelial dysfunction.

Publications arising from 570789:
1) Cho JG, Witting PK, Verma M, Wu BJ, Shanu A, Kairaitis K, **Amis TC**, **Wheatley JR**. Tissue Vibration Induces Carotid Artery Endothelial Dysfunction: A Mechanism Linking Snoring and Carotid Atherosclerosis? *Sleep*. Accepted 18/12/2010.
2) Cho JG, **Wheatley JR**. The association of carotid artery disease with snoring and obstructive sleep apnoea: definitions, pathogenesis and treatment. *Aust Journal of Ultrasound in Medicine*. 13: 27-31, 2010.
Abstracts arising from 570789: 1) Cho J, Witting P, Verma M, Amis TC, Wheatley JR. Snoring-like vibration of the carotid artery reduces tissue cyclic guanosine monophosphate (CGMP).*Respirology* 14(Suppl 1) A85, 2009. **2)** Cho J, Witting P, Verma M, Amis TC, Wheatley JR. Reduction of Endothelial Nitric Oxide Bioavailability in Carotid Arteries Exposed to Snoring-Like Vibratory Energy. *Am. J. Respir. Crit. Care Med.*, 179 (Apr) A3984, 2009. **3)** Cho J-G, Witting PK, Verma M, Wu BJ, Shanu A, Amis TC, Wheatley JR. Impaired Endothelial-Dependent Vasodilatation in Carotid Arteries Exposed to Snoring-Like Vibrations. *Am. J. Respir. Crit. Care Med.*, 181 (Apr) A2466, 2010. **4)** Wheatley JR. Does Snoring Cause Vascular Disease? *Sleep and Biological Rhythms* 8 (Suppl 1) A11, 2010. **5)** Cho J, Verma M, Larcos G, Amis TC, Wheatley JR. Loud Snores Reduce Carotid Blood Peak Systolic Velocity and Arterial Wall Shear Stress. *Am. J. Respir. Crit. Care Med.*, 183 (Apr) A, 2011. **6)** Cho J, Verma M, Narayan J, Larcos G, Amis TC, Wheatley JR. Induced Snoring Reduces Carotid Artery Blood Flow and Wall Shear Stress in an Animal Model. International Symposium of Sleep & Breathing, Barcelona, Apr 2011.

ID: 632597.
Title: The Impact of CPAP Treatment on Carotid Artery Atherosclerosis in Heavy Snoring and Mild Sleep Disordered Breathing.
Chief Investigators: JR Wheatley, TC Amis.

This research proposal focuses on the relationship between heavy snoring with mild SDB and early changes of carotid artery atherosclerosis and is supported by results from our previous study identifying heavy snoring as a risk factor for carotid atherosclerosis, and our animal model studies confirming transmission of snoring vibration energy to carotid artery walls .

Aims: The aim of this study is to further explore the linkage between mild SDB with heavy snoring and early carotid artery atherosclerosis, by demonstrating regression of early changes of carotid atherosclerosis in heavy snorers with mild non-hypoxic SDB, when snoring and SDB are prevented by the use of nasal continuous positive airway pressure (CPAP). The study will achieve this aim by testing for carotid atherosclerosis regression and/or stabilisation (using measures of carotid artery intima-media thickness) with a prospective, randomised controlled study of nasal CPAP in heavy snorers with mild to moderate SDB.

Hypothesis: Early changes of carotid artery atherosclerosis seen in patients with heavy snoring and mild SDB will stabilise or regress following treatment with nasal CPAP therapy.

Progress:
1. This three year trial commenced in February 2010.
2. The study protocol has been registered on the Australian New Zealand Clinical Trials Registry.
3. Progress has been slowed by delays in recruitment of suitable staff to work on the project. This has now been resolved, and work on the trial has commenced.
4. Because of the large number of carotid ultrasounds required for this project, we have had to source our own Ultrasound machine for the duration of the project. This has been completed. We have also had to engage a part-time ultrasonographer to assist with data capture.
5. CPAP masks and machines required for therapy have now been acquired and all necessary equipment is now available for use.
6. Appropriate screening protocols have now been put in place in all our sleep clinics to enable the rapid recruitment of the appropriate cohort of heavy snorers. This includes appropriate collection of anthropometric details and routine measurement of fasting blood glucose and lipids in the clinic.
7. We have undertaken software development of our routine polysomnography sleep studies to enable automatic measurement and calculation of snoring indices, which is critical to identifying the appropriate study cohort.
8. Development of the detailed study protocol and clinical case report form (CRF) has been completed.
9. Screening subjects for the run-in period has commenced, and recruitment and randomisation will continue throughout 2011.

Publications arising from 632597:
There are no publications arising from this study to date. As this is a three year prospective, randomised, controlled clinical study, we do not anticipate any publications arising until completion of the study.
Over the last 12 months there have been 4 publications related to this work:

1. Cho JG, **Wheatley JR**. *Australasian Journal of Ultrasound in Medicine*. 13: 27-31, 2010.
2. Amatoury J, at al. *Journal of Applied Physiology*. 109: 1467-1475, 2010.
3. Kirkness JP, et al. *Physiological Measurement*. Accepted 17/11/2010
4. Cho JG, et al. *Sleep*. Accepted 19/12/2010.

PROGRESS REPORT NHMRC GRANT: 386201
Chief Investigators: Dewey, H; Bernhardt, J; Donnan, GA; Thrift, AG; Carter, R; Lindley, R
Administering Institution: National Stroke Research Institute
Grant Commenced: 1.1.2006
Project Title: Improving outcome after stroke: A large multi-centre, randomized, controlled trial of early mobilization (AVERT).

The goal set by the management team for 2010 was to continue to expand the trial both nationally and internationally to meet a recruitment target of 30+ patients per month. This was achieved in mid 2010. Now 36 hospitals in New Zealand, Northern Ireland, Scotland, Wales, Malaysia, Singapore and Australia participate. A further 7 hospitals in England and Scotland have been trained and will start soon. At the end of 2009 we were successful in obtaining a 3 year grant from the UK Stroke Association (GBP 185,650) to support a full time trial manager. Rosemary Morrison was appointed in early August and received training in Australia in August. Our Australasian trial manager travelled to the UK to support site selection and training of new sites which is now well underway. In 2010, 290 patients were recruited. Data quality has been monitored regularly and is very satisfactory. Follow-up interviews have been occurring as planned and there have been only 5 drop outs from 900 patients (0.5%) to date. The Data Safety and Monitoring Committee met and recommend we continue as planned. We have approval for an extension in time for this project and will use remaining carry forwards funds to support the trial in 2011. An application of funding to support completion of this international trial has been submitted.

Publications (2010)

1. (in press) van Wijks R, Cumming T, Churilov L, Donnan G, Bernhardt J. An Early Mobilisation Protocol Successfully Delivers More and Earlier Therapy to Acute Stroke Patients: Further results from AVERT. *Neurorehabilitation andNeural Repair*
2. (in press) Cumming T, Collier J, Thrift A, Churilov L, Dewey H, Donnan G, Bernhardt J. Very early mobilisation after stroke fast tracks return to walking: Further results from a randomised controlled trial (AVERT). *Stroke*
3. 2010 Janssen H, Bernhardt J, Collier J, Sena E, McElduff P, Attia J, Pollack M, Howells D, Nilsson M, Calford M and Spratt J. An Enriched Environment Improves Sensorimotor Function Post–Ischemic Stroke. *Neurorehabilitation andNeural Repair* (Published online before print September 12, 2010, doi: 10.1177/1545968310372092)
4. 2010 Craig L, Bernhardt J, Langhorne P, Wu O. Early mobilisation after stroke: an example of an individual patient data meta-analysis of a complex intervention. *Stroke:***41:2632-2636.**
5. 2010 Bernhardt J. Sehr frühe Mobilisation nach Schlaganfall [Very early mobilisation after stroke]. Invited review translated into German. *Neuroreha.*4:153-159.
6. 2010 Skarin K, Bernhardt J, Sjoholm A, Nilsson M, Linden T. Better wear out sheets than shoes: A survey of 202 stroke professionals' early mobilisation practices and concerns. *International Journal of Stroke* 6:10-15. 2010
7. Zhao H, Collier JM, Quah D, Purvis T, Bernhardt J. The modified Rankin Scale in acute stroke has good inter-rater-reliability but questionable validity. *Cerebrovascular Dis* 29: 188-193.
8. 2010 Langhorne P, Stott D, Knight A, Bernhardt J, Barer D, Watkins C. Very Early Rehabilitation or Intensive Telemetry after Stroke: A Pilot Randomised Trial *Cerebrovascular Diseases* 29:352-360.

Project Title: INTERACT: a randomised trial of intensive blood pressure lowering in acute intracerebral haemorrhage

Chief Investigators: Anderson C (CIA), Chalmers J, Wang J, Lindley R.
Administering Institution: The University of Sydney
Grant Commenced: 2008

Objectives

The second (main phase) Intensive Blood Pressure Reduction in Acute Cerebral Haemorrhage Trial (INTERACT2) aims to provide definitive evidence that will underpin a potentially new and widely applicable treatment for patients with acute intracerebral haemorrhage (ICH), the most serious form of stroke. The specific primary aim is to define the effects of early intensive blood pressure (BP) lowering (140 mmHg systolic target) compared to current, more conservative, guideline-based BP lowering (180 mmHg systolic level) on the risk of the composite endpoint of death or dependency at 90 days in 2,800 patients with ICH who are able to commence treatment within 6 hours of onset and are otherwise able to receive best usual active medical care. Secondary aims are to assess the effects of treatment separately on death and dependency, and on the need for permanent residential care, quality of life, recurrent vascular events, and length of hospital stay.

Progress

The study continues to progress well. Key milestones for this report include: (a) 1675 randomised patients in the study globally, 80% from China; (b) a successful European Investigator meeting attended by 40 investigators and co-ordinators from 10 European countries at the European Stroke Conference in Barcelona in May 2010; (c) ongoing roll-out of active study sites in Northern Europe, UK and South America; (d) ongoing approval from the independent Data Monitoring Committee to continue recruitment as per their September 2010 meeting (this was their first interim outcome analysis; their next meeting primarily for safety is scheduled for March 2011); and (e) ongoing publications from INTERACT1 listed below. INTERACT2 is now the largest ever clinical trial in ICH to date, and one of the fastest ever recruiting stroke trials (60-90 patients recruited per month).

Relevant publications in 2010/11

1. Morgenstern LB, Claude Hemphill III J, Anderson C, et al. Guidelines for the management of spontaneous intracerebral hemorrhage. *Stroke* 2010;41;2108-29.
2. Anderson CS, Huang Y, Arima H, et al. Effects of Early Intensive Blood Pressure-Lowering Treatment on the Growth of Hematoma and Perihematomal Edema in Acute Intracerebral Hemorrhage. The Intensive Blood Pressure Reduction in Acute Cerebral Haemorrhage Trial (INTERACT). *Stroke* 2010;41:307-12.
3. Delcourt C, Huang Y, Wang J, et al. The second (main) phase of an open, randomised, multicentre study to investigate the effectiveness of an Intensive blood pressure reduction in acute cerebral haemorrhage trial (INTERACT2). *Int J Stroke* 2010;5:110-6.
4. Arima H, Anderson CS, Wang JG, et al. Lower treatment blood pressure is associated with greatest reduction in hematoma growth after acute intracerebral hemorrhage. *Hypertension* 2010;56:852-8.

NHMRC Application ID	**512429**
Administering Institution	**The University of Sydney**
Chief Investigator A	**Dr Maree Hackett,** Glozier N, **Jan S**, Lindley R
Project Title	**Psychosocial Outcomes In StrokE (POISE)**
Year Funding Commenced	**2008**

This multi-centre, three year cohort study will recruit a representative sample of younger (< 65 years) stroke survivors to determine the modifiable predictors of subsequent return to work. Participants will be recruited from the New South Wales Stroke Services (SSNSW) network.

1. The primary aim of this project is to determine if modifiable early (within 28 days of stroke) psychosocial factors are associated with return to work at one year in younger stroke survivors.

2. The secondary aim is to determine the economic impact of not returning to work for younger stroke survivors and their families.

Recruitment commenced in October 2008 and 440 participants were recruited from 20 sites across New South Wales.

Follow-up interviews at 28-days and six months are complete. 12 month follow-up interviews are progressing smoothly with losses to follow-up (for any reason) under 10%. Follow-up interviews are planned to be complete by May 2011.

Other achievements for this study include:

a) Registration of the study protocol on the Australian New Zealand Clinical Trials Registry (ANZCTR): ACTRN12608000311358 in the observational study category,

b) Publication of the study protocol: **Hackett ML**, Glozier N, **Jan S**, Lindley R, Psychosocial Outcomes in StrokE: the POISE observational stroke study protocol. *BMC Neurology* 2009;9:24,

c) Platform presentation of some preliminary baseline results at the Stroke Society of Australasia Annual Scientific Meeting in Melbourne in 2010 with the published abstract: O'Reilly R, Arblaster L, **Jan S**, Glozier N, Lindley R, **Hackett M**. The importance of participant initiated information during an observational study of psychosocial outcomes in stroke (POISE). *International Journal of Stroke* 2010;5(Suppl 1)12.

Genetic Markers for Retinal Arteriolar Narrowing and Risk of Hypertension and Coronary Heart Disease (2008-2010)

CIs: TY Wong, S Harrap, JJ Wang, P Baird. P Mitchell, G Liew.

Aims: The aims of this project are to determine genetic markers for retinal arteriolar narrowing, a novel subclinical vascular phenotype that represents a visible measurable manifestation of early pathological processes in hypertension. Our study uses genomic data from the population-based Blue Mountains Eye Study (BMES) to identify novel gene variants associated with this phenotype, determine how these genetic markers interact with environmental influences in the pathogenesis of microvascular disease, and correlate these genetic markers with the longitudinal risk of incident hypertension and coronary heart disease. This study will improve understanding of the genetic determinants of vascular disease.

Progress: Genotyping on the highest and the lowest quartiles of central retinal arterial equivalent (CRAE) has been completed, as has the GWAS data quality checking and imputation from HapMap in 2009. In addition, we have genotyped the whole BMES sample and imputed SNPs from the 1000 genomes in 2010. Genetic association analyses have been explored using both the case-control and the whole BMES GWAS data. To enhance evidence quality, we have collaborated with the Cohorts for Heart and Aging Research in Genomic Epidemiology (CHARGE) Consortium, which consists of five population-based cohorts, and other population-based studies in the USA, UK and Singapore, to assess genetic associations with CRAE. Initial analyses of the data by individual study were completed and meta-analyses of findings from all studies have also been completed. Two SNPs were found to be associated with CRAE with p values at GWAS significance level. A manuscript is currently under preparation and near completion.

In addition, detailed analyses for genetic associations with CRAE using BMES data are currently ongoing to carefully investigate effects from, and interactions with known cardiovascular disease risk factors on CRAE genetic associations, using different models containing different co-study variables. An abstract has been presented as an oral presentation at the Annual Scientific Meeting of the High Blood Pressure Research Council of Australia, December 2010, Melbourne.

Publications: Articles published in 2008-09 in the relevant area:

1. Sun C, Wang JJ, Islam FM, et al. Hypertension genes and retinal vascular calibre: the Cardiovascular Health Study. *Journal of Human Hypertension* 23:578-84, 2009
2. Wang JJ, Rochtchina E, Liew G, et al.The long-term relationship between retinal arteriolar narrowing, blood pressure and incident severe hypertension. *American Journal of Epidemiology* 168:80-8, 2008
3. McGeechan K, Liew G, Macaskill P, et al. Retinal vessel caliber and prediction of coronary heart disease: A systematic review and meta-analysis. *Annals of Internal Medicine* 151:404-13, 2009
4. Tanabe Y, Kawasaki R, Wang JJ, et al. Angiotensin converting enzyme gene and retinal arteriolar narrowing: The Funagata Study. *Journal of Human Hypertension* 23:788-93, 2009
5. Sasongko MB, Wong TY, Wang JJ. Retinal arteriolar changes: Intermediate pathways linking early life exposures to cardiovascular disease? *Microcirculation* 170:1-11, 2010

Articles generated from this project:

6. Ikram MK*, Xueling S*, Jensen RA*, Cotch MF*, Hewitt AW* (all first authors), *et al.* (CIA is a senior/ last author). Four novel loci (19q13, 6q24, 12q24 and 5q14) influence the microcirculation in vivo. *PLoS Genetics* 6 (10): e1001184, 2010
7. Xueling S*, Jensen RA*, Ikram MK*, Cotch MF* (all first authors), *et al.* (CIA, CIC are last authors) The CHARGE Eye Working group. Genetic loci for retinal arteriolar microcirculation. Manuscript under preparation to be submitted in April 2011.

An abstract presented at international conference:

Wang JJ, Xie J, Inouye M, et al. Genome-wide association study of retinal arterial dimensions and interaction with coronary risk factors. Presented at the 2010 Scientific Annual Meeting of the High Blood Pressure Research Council of Australia, December 2010, Melbourne.

Genes and Environment in the Risk of Early Age-Related Macular Degeneration: A Population-based Case-Control Study (2008-2010)
CIs: JJ Wang, J Attia, PN Baird, WT Smith, P Moscato, R Guymer

Aims: The aims of this project are to: 1) confirm the risk magnitude of current known AMD-associated genetic variants for early and late AMD in a large combined population-based sample; 2) identify new genetic loci associated with early and late AMD; and 3) assess the joint effects of combined genes or genes and environmental factors in predicting risk of AMD. This study will pool AMD case-control samples from three well-conducted, population-based cohort studies of older persons (Blue Mountains Eye Study, BMES, Australia; Beaver Dam Eye Study, BDES, USA and Rotterdam Study, RS, the Netherlands), to investigate joint effects of multiple factors on AMD risk. We will conduct genome-wide association analysis in BMES and RS, replicated in the BDES, and perform meta-analysis to confirm the associated genetic variants. We will then systematically explore possible interactions and joint effects of identified AMD-related gene variants and environmental factors in the pooled datasets. This project will provide a unique international opportunity to advance knowledge of AMD pathogenesis, and to gain insights into the interplay of nature and nurture, and how this affects a critical human disease.

Progress: Genotyping on all AMD cases and controls has been completed, as has the GWAS data quality checking and imputation from HapMap in 2009. In addition, we have genotyped the whole BMES sample and imputed SNPs from the 1000 genomes for the BMES population GWAS data in 2010. Phenotype validation on late AMD across the BMES, BDES and RS (the Three Continent Studies) has been completed in 90% of the cases through regular teleconferences among the study investigators of the three studies. Newly developed late AMD cases in most recent visits will go through the same cross-checking procedures in early 2011. Inter-grader validation on early AMD assessment has also been completed. A standard AMD severity grading scheme has been proposed to be used by all three studies. Project topics for Aim 3 have been allocated to each study team of the three studies and project proposals have been prepared by each of the study teams.

We are planning to replicate these findings in the RS and may also replicate in an international consortium, the Cohorts for Heart and Aging Research in Genomic Epidemiology (CHARGE) Consortium. CIA, CIB and a post doc fellow in the Newcastle University will lead Aim 2 of this project. Analysis plan has been drafted, discussed among the CHARGE groups and will be finalised in February 2011, followed by analyses within each group and then meta-analysis.

An improvement in computation speed for bioinformatics has been achieved by the research fellow under CIE. This improved method was presented in the Biomarker Discovery Conference held in December 2010 in Shoal Bay, NSW. Analyses using bioinformatics methods are ongoing, and significant joint contribution from two or more SNPs is suggested. A number of SNPs found to be possibly associated with early AMD in the BMES data showed consistency across four different analysis methods used. A methodology paper is under preparation by the team in Newcastle University, led by CIE and CIB. These findings are to be replicated in the RS and then the methodology paper will be submitted for publication.

Publications: Following in this research direction, we published two relevant articles using existing BMES data, and one paper in preparation.

1. Wang JJ, Ross R, Tuo J, Burlutsky G, Tan AG, Chan CC, Favaloro EJ, Williams A, Mitchell P. The *LOC387715* polymorphism, inflammatory markers, smoking and risk of age-related macular degeneration: A population-based case control study. *Ophthalmology* 155:693-9, 2008
2. Wang JJ, Rochtchina E, Smith W, Klein R, Klein BEK, Joshi T, Sivakumaran TA, Iyengar S, Mitchell P. Combined effects of Complement Factor H with smoking, dietary fish consumption and inflammatory markers on the risk of age-related macular degeneration in a cohort. *American Journal of Epidemiology* 169:633-41, 2009
3. [Authors to be finalised] Consistency across four analytical methods identifies novel age related macular degeneration loci. To be finalised and submitted in March 2011.

Sydney Adolescent Vascular and Eye Study (2008-2011)

CIs: Paul Mitchell, Wayne Smith, Kathryn Rose, Jie Jin Wang, Victoria Flood, Ian Morgan

Aims: SAVES is a longitudinal study based on the two population-based cohorts examined as part of the Sydney Myopia Study/ Sydney Childhood Eye Study (SMS/ SCES, NHMRC 253732, 2003-2005), totalling 4,093 children. It had two principal but linked components:

1) Longitudinal vascular study examining the genesis of cardiovascular risk in childhood;
2) Longitudinal eye study examining emergence of significant visual impairment, causes, refractive error change (including early myopic retinal signs), eye parameters & determinants.

SAVES aims to correlate 5-year retinal microvascular changes during adolescence with baseline measures of retinal vessel calibre & current risk factors for cardiovascular disease. Identifying early microvascular changes in children with higher blood pressure (BP) levels, obesity, low birth weight or premature birth, will improve understanding of how CVD and its risk factors/components, such as hypertension, develop into adulthood. SAVES will also document longitudinal changes in eye health, using sophisticated digital ocular scanning instruments to measure both ocular biometry parameters & optical coherence tomography. The causes of myopia and early myopic retinal degenerative signs, particularly in relation to risk factors such as educational achievement, East Asian ethnicity & variable outdoor activity, remain key unresolved questions. SAVES will assess longitudinal changes in eye health (myopia; both mild or high levels, and associated subtle myopic retinal changes, plus amblyopia and strabismus, in relation to risk factors like education & ethnicity.

Progress: During 2009-10, 17/18 high schools originally involved were visited, with a single Catholic school uninterested in a re-visit. The response rate from previously tested adolescent participants currently at these schools is over 80%. Of 34 primary schools originally involved, most were re-visited. Some schools were not able to be re-visited before students left Yr 6, so we have visited feeder schools (or high school classes in the same school) for Yr 7-8 students. Some of the remaining (mainly Catholic) schools are currently being attended in Term 1; data collection should be complete late April 2011. To date, 2760 children have been tested. We also traced most children who left their initial school; many were seen in other schools, some are still to be examined at Westmead Hospital Eye Clinic. Correspondence to all baseline participants and their parents has been sent, and data entry of the detailed examination and questionnaire books of all children seen to date is now largely complete. Data from the various instrument outputs have been cleaned, and are currently in analysis. Retinal vascular assessment of retinal photos is well underway, including sophisticated retinal vessel and fractal grading measures, and assessment of other retinal lesions.

Publications:

1. Gopinath B, Baur LA, Wang JJ, *et al*. Blood pressure is associated with retinal vessel signs in preadolescent children. *J Hypertens* 28:1406-12, 2010
2. Leone JF, Mitchell P, Morgan IG, Kifley A, Rose KA. Use of visual acuity to screen for significant refractive errors in adolescents: is it reliable? *Arch Ophthalmol* 128:894-9, 2010
3. Gopinath B, Baur LA, Wang JJ, *et al*. Smaller birth size is associated with narrower retinal arterioles in early adolescence. *Microcirculation* 17: 660-8, 2010
4. Tariq YM, Samarawickrama C, Li SH, Huynh SC, Burlutsky G, Mitchell P. Retinal thickness in the offspring of diabetic pregnancies. *American Journal of Ophthalmology* 150: 883-7.e1, 2010
5. Gopinath B, Baur LA, Teber E, Liew G, Wong TY, Mitchell P. Effect of obesity on retinal vascular structure in pre-adolescent children. *Int J Ped Obesity* 1 Oct [Epub ahead of print], 2010
6. Tariq Y, Gole G, Burlutsky G, *et al*. Association of birth parameters with OCT measured macular and retinal nerve fibre thickness. *Inv Ophthalmol Vis Sci* 6 Jan [Epub ahead of print], 2011
7. Gopinath B, Baur LA, Mitchell P, *et al.* Influence of physical activity and screen time on retinal microvasculature in young children. *Arteriosclerosis, Thromb Vasc Biol* in press, 24 Jan, 2011
9. Gopinath B, Hardy L, Teber E, *et al.* Association between physical activity and blood pressure in pre-pubertal children. *Hypertension Research* in press, 8 February, 2011
10. Gopinath B, Baur LA, Mitchell P, et al. Relationship between a range of sedentary behaviors and blood pressure during early adolescence. *J Hum Hypertens* in press, 14 March, 2011.

Genetic associations of early retinal pathologic phenotypes: Data pooling and meta-analyses of multiple populations (2010-2012)

CIs: JJ Wang, E Boerwinkle, G Liew, P Moscato, S Tai, A Hewitt. J Attia, YY Teo, R Klein, P McElduff.

Aims

This project focuses on two types of retinal phenotypes: early vascular retinopathy and early age-related macular degeneration (AMD), and has two aims:

Aim 1: Discover novel gene variants and single nucleotide polymorphisms (SNPs) associated with each retinal phenotype of early vascular retinopathy and early AMD through GWAS. This will be conducted at individual GWAS level in white populations first (the Blue Mountains Eye Study population and studies under the Cohorts for Heart and Aging Research in Genomic Epidemiology (CHARGE) Consortium: the Atherosclerosis Risk in Communities (ARIC) Study and the Cardiovascular Health Study (CHS) in the USA, and the Rotterdam Study (RS) in the Netherlands), with replication in Asians (Malays and Chinese), followed by a meta-analysis, using both traditional statistical approaches as well as a bioinformatics approach.

Aim 2: Explore gene-environment interactions or combined effects of selected gene loci or polygenic clusters with major environmental and systemic factors involved in the pathophysiology of these two early retinal disorders, using both traditional statistical approaches as well as bioinformatics approaches. We will investigate major environmental exposures that have been found to be associated with these retinal phenotypes while controlling for age. These include blood pressure and plasma glucose levels for early retinal vascular retinopathy phenotypes, smoking, inflammatory markers (white cell count), major dietary factors including antioxidants, omega-3 fatty acids and glycaemic index for early AMD phenotypes.

Progress

For Aim 1, GWAS of early vascular retinopathy phenotypes have been completed by each of the six studies, as have the meta-analyses. A manuscript on this first phenotype (retinopathy) of Aim 1 is under preparation.

GWAS of early AMD phenotypes have been conducted in the BMES sample, from which we have indentified some consistent signals (SNPs) across the three or four different methods we used. A methodology paper about the use of multiple methods to detect consistency in the association is ready for submission for pulication. An analysis plan for GWAS on early AMD phenotypes has been circulated among the CHARGE studies, and analyses and meta-analyses of the findings from all the studies will be conducted in 2011.

Analyses for Aim 2 will commence soon after the significant SNPs are identified for each phenotype.

Publications

There are no publications at this stage.

CIA: Professor John R Wheatley MBBS (Hons), FRACP, PhD

John Wheatley is a Professor of Medicine in the Sydney Medical School, University of Sydney, and Director of the Ludwig Engel Centre for Respiratory Research in the Westmead Millennium Institute. He is also Director of the Department of Respiratory and Sleep Medicine at Westmead Hospital, and a Consultant Respiratory and Sleep Medicine Physician at Westmead Hospital. His research has been in the area of the pathophysiology of upper airway function in relation to snoring and the obstructive sleep apnoea syndrome.

A. Publications:
-68 refereed Journal articles reporting original research, mainly in top ranking Sleep and Respiratory specialty Journals. Over 180 conference abstract presentations.

B. Grants and Fellowships:
-NHMRC Practitioner Fellowship 2010-2015
-14 past and 2 current NH&MRC project grants as Chief Investigator (7 as CIA). 12 national grants from specialist agencies and 13 grants from other sources, all as Chief Investigator. Continuous NH&MRC project grant funding since 1992.

C. Peer Recognition: Invitations to Speak:
-Invited speaker at 19 international meetings, including 13 plenary sessions at major international meetings and invited speaker at 6 other international meetings.
-Eleven plenary presentations as an invited speaker at major national meetings and five other national presentations. Session chairing at international meetings on 14 occasions, and multiple occasions at local national meetings.

D. Editorial Positions
-Editorial Board member *Sleep* (2003-11)
-Associate Editor (2008-11) of *Sleep* (International Specialist Journal)

E. Prizes and Awards:
-New South Wales Department of Health Medical Research Scholarship (1987)
-NH&MRC post-graduate medical research scholarship (1988-89)
-Medical Foundation Travelling Fellowship (1991-92)
-Faculty of Medicine post-graduate travelling scholarship, University of Sydney (1991-92)
-John Read Prize for Physiological Research – TSANZ and ALF (1997, 1998, 2006; shared)
-Australasian Sleep Association **Distinguished Achievement Award, 2009**

F. Research Translation: Clinical Activities:
-International clinical leadership as group leader of a committee which established clinical practice guidelines for polysomnography studies (both research and clinical), and lead to a change in clinical conduct of sleep studies around the world.
-International leadership as **Co-Chairman of worldsleep07**, the World Sleep Congress for WFSRSMS held in Australia for the first time in September 2007 (attendance 2,300). Only held every four years, this is the largest and most prestigious Sleep Conference held in the world outside of the USA, which has substantially enhanced Australia's profile internationally among professional sleep organizations.
-Director of LECRR, one of the largest respiratory sleep research teams in Australia where research is successfully combined with clinical practice. Lead role in national clinical trial research.

-Director of the Department of Respiratory and Sleep Medicine, Respiratory Failure and Sleep Disorder Services at Westmead Hospital, with a national profile for clinical practice and research.
-National leadership in professional development of Sleep Medicine, including Sleep Medicine Curriculum development, and sleep and respiratory physician training programs.

G. Contributions to Research Training:

-Primary supervisor of five successfully completed PhD candidates, with a further three currently enrolled PhD candidates. Successful completions of one Masters candidate and eight Honours research students.
-Training of three post-doctoral staff, and more than 20 general research staff.

H. Contributions to Professional Activities:

-Member of NH&MRC discipline panel for physiological sciences (1999, 2000)
-Member of Research Advisory Committee for Asthma Foundation of NSW (1996-2003)
-Reviewer for 18 NH&MRC project grants, and 57 grants for other national and state organizations
-President (1997-9), Honorary Secretary (1994-7), and Executive Committee Member (1992-2000) for Australasian Sleep Association
-Executive Committee Member for TSANZ (1998-2000)
-Chairman, SAC in Respiratory and Sleep Medicine (RACP/ASA) (2000-8)
-Chairman, Sleep Curriculum Development Committee (ASA/RACP) (2008-10)
-Governing Council Member, WFSRSMS (1999-2007)
-Co-Chairman Organising Committee for WFSRSMS Congress, Cairns (2007)
-American Thoracic Society Program Committee for Resp Neurobiology and Sleep (2004-5)
-Member of organising committee for 11 national meetings and three international meetings.

I. Most Significant Scientific Contributions

1) Development of a device and process for the measurement of surface tension in UA liquid, unique to our laboratory, which resulted in collaborative requests from Adelaide and Perth, and international groups in Baltimore (USA), Quebec (Canada), and Japan.
2) In 1997, participated as international task force group leader for the ASDA, establishing the methods and standards for research and clinical PSG which are now in use through the world, resulting in a seminal publication with a citation index of 1184.
3) Described the loss of negative pressure reflex activation of the genioglossus muscle during sleep, substantially advancing our understanding of the pathophysiology of OSA and leading to treatment trials of electrical stimulation of upper airway muscles during sleep.
4) In a recent publication from our group, was the first to demonstrate that snoring per se is a risk factor for carotid atherosclerosis, separate to OSA and hypoxia. This has significant implications for the management of snoring in relation to prevention of stroke. The paper was the subject of two separate editorial commentaries in the journal *Sleep*.

RICHARD LINDLEY

Positions: The Moran Foundation for Older Australians, Professor of Geriatric Medicine, University of Sydney (since 2003), and Professorial Fellow, The George Institute for Global Health (since 2010). Head, Discipline of Medicine, Western Clinical School, The University of Sydney (2006-10). Consultant Physician and Geriatrician and Senior Lecturer at the University of Edinburgh, Scotland (1996-2003).

Awards: British Geriatric Society: Norman Exton-Smith prize (1992), Woodford Williams prize (1993), Eric Dreikurs Heart Foundation (Australia) Research Award (2005) Westmead Hospital Excellence in Teaching (Consultant) 2004/5, Honorary Foreign Member of the Association of British Neurologists (awarded 2008)

Research: Professor Lindley's broad research interests concentrate on collaborative research in clinical medicine (geriatric medicine, frailty, stroke and randomised controlled clinical trials). He has been a co-investigator for many of the RCTs that have changed clinical practice for older people, such as aspirin for acute ischaemic stroke (IST Lancet 1997 paper 830 cites), blood pressure lowering for stroke secondary prevention (PROGRESS Lancet 2001 paper 1167 cites), statins for the high risk elderly (HPS Lancet 2002 paper 3504 cites), feeding regimes after acute stroke (FOOD Trial Lancet 2005 paper 93 cites), dual antiplatelet therapy for stroke secondary prevention (ESPRIT Lancet 2006 paper 278 cites), anti-emboli stockings for patients with stroke (CLOTS Lancet 2009 paper 29 cites) and currently thrombolysis for stroke (The Third International Stroke Trial: IST-3). His work on lacunar small vessel disease and retinal microvascular changes led to a Lancet Neurology publication in 2009, with accompanying editorial and personal feature (Lifeline), and has subsequently led to invitations to teach at the European Stroke Conference in 2010 and planned for 2011. He was the first to involve consumers in the design of a stroke RCT (BMJ 2002 paper 47 cites) that has been widely used as a "best practice" example in consumer literature. He is currently the Principal Investigator or Grant holder in the world's largest trials of: thrombolysis for stroke (IST-3); acute treatment (blood pressure lowering) for haemorrhagic stroke (INTERACT-2); and early and intense rehabilitation for acute stroke (AVERT). To date he has been (or is currently) an investigator in 29 RCT's ranging from breast cancer treatment to the prevention of falls.

Research Grants: He has raised $20,000,000 in research grants in his career (about a third of that sum was raised in the UK for the IST-3 project prior to arriving in Australia). Half of the total research funding was obtained as CIA. These large sums mainly represent the funding for national and international large scale RCTs in Stroke and Geriatric Medicine.

Invited presentations: 79 invited lectures in ten countries in the past decade, with an emphasis on evidence based medicine, geriatric medicine, frailty, disability, randomised controlled trials and stroke**.**

Teaching: Lecturer at clinical trial courses in London and Edinburgh, and current major teaching commitment to the Graduate Medical Program of the Sydney Medical School, University of Sydney that includes lectures (whole of year and recorded web –based), beside ward teaching (geriatric and stroke medicine) and he leads the Evidence Based Medicine Course at Sydney Medical School – Westmead (PEARLS).

Theses supervised: One completed Doctor of Medicine (as primary supervisor), two completed Doctor of Medicine (as associate supervisor) one completed Master of Medicine, 3 completed honours students and 1 current postgraduate student.

Publications: 161 publications (128 peer reviewed), including two books on Stroke ("Stroke The Facts". Oxford University Press 2008. ISBN 978-0-19-921272-9 and "Understanding Stroke". The British Medical Association Family Doctor Series. ISBN: 1 898205 60 4. Published by Family Doctor Publications Ltd in association with the British Medical Association) and four chapters.

Reviews: A regular reviewer for numerous journals including the BMJ, Brain, Age and Ageing, Lancet Neurology, Lancet, Stroke, Annals of Neurology and governmental agencies including the NHS (UK), the NHMRC, the National Heart Foundation, the New Zealand Health Research Council, National Medical Research Council of Singapore.

Committee memberships and other positions: Member, National Stroke Foundation Acute Stroke Guidelines (2005-7); Member, Stroke Rehabilitation and Recovery guideline committees (2004/5); Director National Stroke Foundation (appointed 2005 - present); Chairman of the Scottish national stroke guideline committee (SIGN) (1999-2002); Member, Scientific Committee for the British Geriatrics Society (2000-2002); Education and Training Committee for the Australian and New Zealand Society for Geriatric Medicine; Chairman of the Research Sub-Committee of Stroke Services NSW (2004-present). Associate Editor for Australasian Journal for Ageing (2006 to present); Stroke Society of Australasia Honorary Secretary (2005-7), Vice-President (2007 to 2010), President (2010 to present). Co-chairman of GMCT NSW Aged Care Network (2007-10). Chairman of 3 Data Monitoring Committees (CSL vaccine trials 2008-10). Outcome Committee member for three RCTs.

Research Career and Scope of Topics

Associate Professor Jie Jin Wang is a key investigator on one of the landmark Australian population-based cohort studies, the Blue Mountains Eye Study (BMES), and has been the principal researcher for many major BMES sub-studies, including assessments of the 5- and 10-year incidence and progression of age-related macular degeneration (AMD) studies examining the relation of retinal vessel signs and systemic outcomes, and projects that have pooled BMES data with data from the US Beaver Dam Eye Study (BDES). Jie Jin has also initiated a number of international and national collaborations that are extending the scope of the BMES cohort study. She has generated, conceptualised and led broad-based research programmes and initiated collaborations with researchers outside the Centre for Vision Research. Examples of her initiatives that developed into research proposals and gained NHMRC funding are IDs 352337, 475604 and 512423. The last (512423) is a large-scale project to pool phenotype and genotype data from 3 population-based studies (BMES, BDES, and the Rotterdam Eye Study) to investigate the interplay of genetic and environmental factors in determining the risk of AMD.

One of her major research areas has been on the link between the retina and the brain and other systemic conditions. She is CIC on project ID 352337 on the associations of retinal vascular signs with acute stroke subtypes. Findings from this project contribute to the understanding of likely mechanisms of various acute stroke subtypes, with 9 articles published or in press in *The Lancet Neurology*, *Annals of Neurology, Archives of Neurology* and *Stroke,* and another 3 either submitted or in preparation.

Since 2006, when Jie Jin commenced a conjoint appointment as Associate Professor in the Centre for Eye Research Australia (CERA), University of Melbourne, her research leadership role has expanded to the Retinal Vascular Imaging Centre (RetVic) team, led by Prof Tien Y Wong. She has contributed significantly to the development of new projects in this team (475604, 475617 and 590204). Project 590204 involves an international collaboration with the Cohorts for Heart and Aging Research in Genomic Epidemiology (CHARGE) Consortium.

Jie Jin is expanding her research to the focus of the interplay of genetic and environmental factors that contribute to common complex ocular and systemic conditions. She has developed two successful NHMRC project grants (512423, 590204) in this direction in the past few years, and is applying for funding to support her other two new projects. One of the new projects is to use nutrigentic approaches to assess factors contributing to high cardiovascular disease (CVD) risk profiles, and identify patterns of the interplay, in order to develop and implement interventions targeting modifiable factors (such as dietary) for improvement in population health. Another new project is to assess the combined influences of genetic and environmental factors on age-related cataract development, the most common eye disease affecting vision in older persons, and demanding substantial Medicare costs for surgical treatment of this common condition.

Research Support and Output

Jie Jin has been awarded NHMRC Senior Research Fellowship funding in two consecutive funding periods, Level A for 2005-9 and level B for 2010-4. Her research has attracted over $3 million from NHMRC and other granting bodies for projects on which she is lead investigator, and a total of over $15 million for projects on which she is a key co-investigator. She has 233 in print publications (246 including e-publications) since January 2006, and has been invited to contribute to editorials and reviews in peer-reviewed journals such as *Hypertension, Expert Review of Ophthalmology* and *American Journal of Ophthalmology.* As lead author, she has also contributed to three book chapters on the epidemiology of AMD for books titled "Retinal Degenerations" and "Clinical Maculopathy" (in Chinese), and on epidemiology and clinical research for "Principles of Epidemiology". Jie Jin completed two contract literature review projects for the NHMRC ('Management of Diabetic Retinopathy' and 'Diabetes and Day Surgery'), and was joint technical writer for the first version of 'Clinical Practice Guidelines: Management of Diabetic Retinopathy'

(1996).

Postgraduate Supervision

Jie Jin has been the primary supervisor for 4 Masters (all completed) and 9 PhD candidates (3 completed), and associate supervisor for two Masters (all completed) and 11 PhDs (8 completed to date). She has also supervised a number of visiting PhD candidates and research fellows from overseas to study in either the Centre for Vision Research (CVR) in Sydney or in CERA in Melbourne. Among these, one PhD candidate from Japan spent 1.5 years in CERA and another PhD candidate from China spent 1 year in CVR. Both candidates had first-authored articles published in, or accepted for publication by, international eye journals while they spent time in Australia and received supervision primarily from Jie Jin. Jie Jin has examined PhD and Masters theses for candidates from the Universities of Sydney, Melbourne, Newcastle and Adelaide in Australia and from the National University of Singapore as well.

Service to Research Committees

Jie Jin has been a member of the Association for Research in Vision and Ophthalmology (ARVO) since 1997, served as a program member for the Clinical Epidemiologic Research Section of ARVO (2003-05) and chaired the program committee (2005). She is an external advisory committee member on a number of research projects conducted overseas. She serves the University of Sydney research community as an Academic Board nominee since 2006, and a postgraduate study coordinator in the Department of Ophthalmology since 2008.

She has been a reviewer for nearly 50 national and international peer-reviewed journals, a guest section-editor, an editorial board member for *Clinical & Experimental Ophthalmology,* since 2001, and a designated reviewer for an editorial series in the *American Journal of Ophthalmology* during 2009-2010. She has been an editorial member for the *Journal of Ophthalmology* since 2009. In 2010, Jie Jin was appointed a member of the International Advisory Panel for *Archives of Ophthalmology*.

Since 2002, Jie Jin has been an external reviewer for many national and international research grant funding bodies, such as the NHMRC (for project and program grant applications) and Diabetes Australia Research Trust in Australia, the Wellcome Trust, Medical Research Council and the BUPA Foundation Research in the UK, and The InZicht Program of ZonMw project grants in the Netherland.

International Profile and Peer Recognition

Jie Jin has been an invited speaker in a number of international conferences: including Asia ARVO (2005, 2007 and 2011), the World Congress of Ophthalmology (2008), the Pacific Academy of Ophthalmology annual meetings (2009, 2010 and 2011). She has been frequently invited to chair presentation and symposia sessions in these meetings, and has been invited to be a session moderator in nearly every year from 2003-2010 at ARVO annual meetings in Fort Lauderdale, Florida, USA. She was an organiser for symposium sessions in the 2011 Pacific Academy of Ophthalmology Annual Meeting in Sydney, and was a member of the International Advisory Committee and the Scientific Program Subcommittee for 2011 Asia ARVO, and also a member of the abstract review panel for the Clinical and Epidemiologic Research category for this Asia ARVO meeting.

Associate Professor Terence C Amis BVSc (Hons), PhD is a Principal Research Fellow in the Faculty of Medicine, University of Sydney, Principal Hospital Scientist at Westmead Hospital, Westmead, NSW and Associate Director of the Ludwig Engel Centre for Respiratory Research in the Westmead Millennium Institute. After a career in academic veterinary medicine (University of Queensland; University of California, Davis) and research training (PhD) with Prof JMB Hughes at the Royal Postgraduate Medical School, University of London, A/Prof Amis has pursued a 23-year, full-time, career in medical research with major research interests in respiratory physiology, upper airway mechanics, and obstructive sleep apnoea (OSA).

Career Pathway: *Academic Veterinary Medicine*: BVSc (First Class Hons), Univ. of Queensland, 1970. Academic (clinical, teaching and research) appointments at Univ. of Queensland, Australia (1970-75), Univ. of Saskatchewan, Canada (1975-76), Univ. of California, Davis, USA (1979-88).

Medical Research: PhD (1979), University of London. Research Fellow (1976-79), Royal Postgraduate Medical School, Hammersmith Hospital, London. Research officer (1988-90)/Senior Research Officer (1990-9)/Senior Hospital Scientist (1999-2008), Principal Scientific Officer (2008-present) Westmead Hospital, Sydney. Associate Director Ludwig Engel Centre for Respiratory Research (2001-Present), Westmead Millennium Institute, Westmead Hospital. Senior Research Fellow (1999-2007)/Principal Research Fellow (2007-2009), Associate Professor (2009-present) Univ. of Sydney.

Publications: Author/co-author: 81 peer reviewed scientific papers, 3 review articles, 4 book chapters, 3 theses and 191 conference presentations. A/Prof Amis is first or senior author on 73% of his publications (h index 17), >95% of which have appeared in major international journals, including 25 publications in the prestigious *Journal of Applied Physiology*, the leading international journal in respiratory physiology research. Twenty-nine of A/Prof Amis' publications have 15 or more citations, and his work is cited an average 28 times per year.

Grants: Chief Investigator on 13 NHMRC Project Grants and 17 other grants since 1990.

Postgraduate Students/Staff Supervision: Supervisor/co-supervisor: 9 PhD, 5 BSc Hons, 2 Masters and 18 undergraduate research project students. Currently, A/Prof Amis provides research leadership, supervision and line management for 7 research staff members and 3 PhD students.

Awards: Captain James Cook Travelling Scholarship, University of Queensland (1975), Parker B Francis Foundation Fellowship, USA (1979), Australian Lung Foundation Ludwig Engel Grant-in-Aid for Physiological Research (1997), John Reid Prize for Physiological Research, Thoracic Society of Australia & New Zealand (1997 [first author], 1998 [co-author] & 2006 [co-author]).

Learned Societies: *Present membership:* Thoracic Society of Australia and New Zealand (member Education & Research Committee 2004-8); *Past memberships:* Australian Veterinary Association, American Thoracic Society, American Physiological Society, Comparative Respiratory Society.

Reviewer: NHMRC Australia (external assessor for >15 years; member Grant Review Panel 2006-7), Community Health & Anti-Tuberculosis Association, Pharmacia Foundation, American Journal of Veterinary Research, Journal of Am Vet Med Assoc, Journal of Applied Physiology, Journal of Sleep Research, Respirology

Major Administrative Positions: Associate Director, Ludwig Engel Centre for Respiratory Research 2001-present; Chair, Westmead Millennium Institute & Research Centres Science Council 2007-9.

Contributions to Research (1994-2010):

- ***Established anaesthetised dog model for studying laryngeal and soft palate muscle*** functional effects on upper airway airflow mechanics. Includes original studies on cricothyoroid, hyoepiglotticus, tensor palatini, levator palatini and musculus uvulae.
- ***Described mechanisms of oro-nasal airflow partitioning*** in both dogs and humans. These studies included some of the first studies of oral airway airflow mechanics.
- ***Examined the functional characteristics of nasal dilator strips*** establishing the geometric and functional effects on the airflow mechanics of the nasal passages.
- ***Described for the first time the breathing route employed by asthmatic subjects*** showing that asthmatics breathe orally during an acute exacerbation thereby exposing the lower airways to cold, dry, unfiltered inhaled gas. These studies formed a successful PhD thesis (M Hallani).
- ***Directed and supervised studies that developed new methodology for measurement of the surface tension of upper airway lining liquid and demonstrated decreased collapsibility and easier re-opening of the upper airway with surface tension lowering agents*** instilled into the pharynx of rabbits, healthy humans and sleeping OSA patients. These studies formed a successful PhD thesis (J Kirkness). Currently research in this area is focused on effects of mouth breathing and the use of exogenous surfactant to reduce the severity of sleep disordered breathing (PhD student: J Chien).
- ***Developed a rabbit model for studying the upper airway extra-luminal tissue space*** **and** demonstrated the role of tissue pressure in the control of upper airway patency. Studies formed a successful PhD thesis (K Kairaitis).
- ***Designed and supervised studies in an animal model and healthy human subjects demonstrating that snoring is associated with depressed arterial baroreflex sensitivity.*** These studies form a completed successful PhD thesis (J Narayan) and show that effects of snoring on baroreflex sensitivity are mediated via associated changes in intra-pleural pressure.
- ***Supervised studies investigating the relationship between surface cephalometric variables and obstructive sleep apnea*** *(*Master's thesis: R Perri).
- ***Modeling the peri-pharyngeal tissue space.*** This project involves development of a finite element analysis model, imaging studies in an animal model and bench model studies in a collapsible tube (PhD student: J Amatoury).
- ***Chief Investigator in research establishing heavy snoring as a risk factor for carotid atherosclerosis in humans.*** This study provided the first evidence that snoring per se is a risk factor for carotid atherosclerosis.
- ***Demonstrated transmission of snoring vibrations to the carotid arteries of rabbits*** as part of hypothesis testing that snoring may be implicated in atherosclerotic plaque rupture and stroke
- ***Established animal model for testing effects of snoring like vibration on carotid artery endothelial function.*** Supervised studies that confirmed endothelial dysfunction in the carotid artery after exposure to a 6hr 60Hz vibration stimulus dose ranged to vibration energy levels encountered in snoring (PhD student: JG Cho).

Dr Kristina Kairaitis (CIE)

BSc (Hons Class 1), MB BS (Hons Class 2 Division 2), FRACP, PhD.

Dr Kristina Kairaitis is an NHMRC Health Professional Training Fellow (60%) at the Ludwig Engel Centre for Respiratory Research, Westmead Millennium Institute, a clinical senior lecturer at the University of Sydney and qualified sleep and respiratory physician in the Department of Respiratory and Sleep Medicine at Westmead Hospital. Her major research interests are in the vascular consequences of obstructive sleep apnea and upper airway physiology in particular peri-pharyngeal tissue mechanics. She is internationally recognised for her original and unique research in upper airway extraluminal tissue pressure and its influence on upper airway patency. She is currently working on understanding the mechanical processes underlying upper airway closure, for which she was recently awarded a prestigious NHMRC Health Professional Training Fellowship.

Key contributions to Research

Dr Kairaitis has made a number of unique, high impact and original contributions to the field of sleep and upper airway physiology which have been published in high impact journals, been the subject of editorials, have won prizes and invitations to speak at international conferences. These include:-

- ***Demonstrated the association of snoring with carotid artery sclerosis*** (KK421**) .** This publication has attracted editorial commentary (see Sleep 31;1204-1205, Sleep 31:1335-1336) and identified as an important paper at the international conference of the American Thoracic Society clinical year in review 2009. It has been cited 23 times since publication in 2008.

- ***Demonstrated in an animal model the induction of carotid arterial endothelial dysfunction in response to snoring like vibrations*** (KK427). This article has been accepted for publication in Sleep Dec 2010. Endothelial dysfunction is the precursor of atherosclerosis, and this unique and groundbreaking research suggests that snoring vibrations may not just be noise pollution but may contribute to the pathogenesis of atherosclerosis.

- ***Demonstrated transmission of intra-pharyngeal vibrations to the lumen of the carotid artery*** (KK 424). This has been identified as a paper of specialist interest in a recent review of morbidity due to OSA (see Curr Opin Pul Med 14:530-536, 2008).

- ***Developed a rabbit model for studying the upper airway extraluminal tissue space*** and demonstrated the role of this pressure in determining upper airway patency. This has attracted editorial commentary (Schwartz *et al* Journal of Applied Physiology 100:5-6, 2006), and an invited presentation at the Annual Scientific Meeting of the Japanese Respiratory Society in Chiba, Japan, 2005. The description of the effect of tracheal traction on tissue pressures (KK423) has been recommended in the Faculty of 1000 Medicine, identified in a review of upper airway function in OSA as a paper of specialist interest (Curr Opin Pulm Med 14:519-524, 2008) and cited 17 times in the last 4 years. This work has resulted in 5 publications (KK418, 419, 423, 425 and 1 below) and a recent published hypothesis (KK426) concerning the function of the pharyngeal muscles and also has been presented in abstract form. Currently we are developing a finite element model of the pharyngeal airway in order to understand further mechanisms underlying airway patency which has been presented in abstract and led to the development of a recently published bench model of the upper airway (KK417)

- ***Described the influence of cholinergic activity on salivary flow rates and surface tension*** and their relationship to upper airway patency (KK420).

Track Record Indicators

Publication record: 12 peer reviewed articles (and 1 in press), 2 of which have been selected for editorial commentary, 1 editorial, 1 review, 1 letter and 28 conference abstracts. One article has been cited 23 times since publication in 2008, and 5 publications have citation rates of 15 or greater, H–index 7. The majority of publications are in specialised highly regarded journals in the field.

Grants and Fellowships: NHMRC postgraduate fellowship (1999-2003), NHMRC Health Professional Training Fellowship (60%) (2010-2015), JT Tweedle Part time Research Fellowship RACP (2006), CID on 1 NHMRC grant, AI on one NHMRC grant, CIA on 3 grants from other philanthropic bodies.

Invitations to speak: Invited speaker at the 45th Annual Scientific meeting of the Japanese Respiratory Society in Chiba, Japan

Prizes, Awards, Honours: BSc with First class Honours in physiology, UNSW 1988 (supervisor Professor Eugenie Lumbers). MB BS, Honours Class 2, Division 2, UNSW 1992. PhD (Medicine, University of Sydney, 2005) 8 prizes during the course of doctoral studies, including one international award for original and unique research into upper airway extraluminal tissue pressure and its influence on upper airway patency, the Anne Elizabeth Suratt Young Investigator Award.

Peer Review: *Journal Invited Reviews*: Journal of Applied Physiology, Sleep, Journal of Sleep Research, Sleep and Biological Rhythms, Medical Engineering and Physics. *Grant Reviews*: Institute of Breathing and Sleep, Austin Hospital Melbourne; Scientific Advisory Committee, Western Sydney Area Health Service.

Professional Association: Panel member, Sleep and Respiratory Neurobiology Organising Committee for the International Scientific Meeting of the American Thoracic Society, 2010-current. Session Chair at International Meeting 2011. Member of the organising committee of the Australasian Sleep Association 2010- current. Session chair 2010 and 2011. Membership: Royal Australasian College of Physicians, Thoracic Society of Australia and New Zealand, Australasian Sleep Association, American Thoracic Society.

Clinical Activities and Responsibilities: Fractional Staff Specialist in the Department of Sleep and Respiratory Medicine, Westmead Hospital. Responsible for inpatient and outpatient care of patients with sleep disorders and respiratory diseases including tuberculosis. Supervision of registrars and residents.

Supervision and/or Mentoring: mentoring 4 PhD students and one masters student since 2006.

Track Record: 3 period of maternity leave Dec 1999-July 2000, September 2002-April 2002, July 2007- February 2008. Worked part time since 2000, and the following publications should also be included:

1. Kristina Kairaitis, Radha Parikh, Rosie Stavrinou, Sarah Garlick, Jason P Kirkness, John R Wheatley and Terence C Amis. Upper airway extraluminal tissue pressure during breathing in rabbits. *Journal of Applied Physiology,* 95(4) 1560-6, 2003.

2. Jason P Kirkness, Hugo K Christenson, Sarah R Garlick, Radha Parikh, **Kristina Kairaitis**, John R Wheatley and Terence C Amis. Decreased surface tension of upper airway mucosal lining liquid increases upper airway patency in anaesthetised rabbits. *Journal of Physiology,* 547(Pt 2): 603-11, 2003.
